# Further Studies on Incomplete Carcinogenesis: Triethylene Melamine (T.E.M.), 1,2-Benzanthracene and β-Propiolactone, as Initiators of Skin Tumour Formation in the Mouse

**DOI:** 10.1038/bjc.1955.14

**Published:** 1955-03

**Authors:** F. J. C. Roe, M. H. Salaman


					
177

FURTHER STUDIES ON INCOMPLETE CARCINOGENESIS:

TRIETHYLENE MELAMINE (T.E.M.), 1,2-BENZANTHRACENE

AND ,3-PROPIOLACTONE, AS INITIATORS OF SKIN

TUMOUR FORMATION IN THE MOUSE.

F. J. C. ROE AND M. H. SALAMAN.
From the Cancer Research Department,

London Hospital Medical College, London, E.1.

Received for publication December 18, 1954.

IN previous papers (Salaman and Roe, 1953; Roe and Salaman, 1954) it was
shown that the application of urethane (ethyl carbamate) to the dorsal skin of
mice followed by repeated applications of croton oil gave rise to many epidermal
tumours, some of which were maJignant,* and that the number of tumours
which appeared was roughly proportional to the total dose of urethane applied.
Urethane alone, applied repeatedly in large doses, produced no tumours, nor any
recognisable histological change in the skin. Repeated application of croton
oil alone produced very few tumours. Repeated application of urethane
following a single "initiating" dose of a carcinogenic hydrocarbon failed to
produce any skin tumours.

The object of these experiments was to test the hypothesis that there exist
"incomplete carcinogens" which act as "initiators" but not as "promoters"
of carcinogenesis. The meaning of these terms was defined. It was concluded
that urethane was such an incomplete carcinogen for mouse skin.

2-Acetylaminofluorene, p-dimethylaminoazobenzene (butter yellow), and
NN-di-(2-chloroethyl)-f,-naphthylamine (R48), were tested by a similar technique,
but none of these showed any initiating power. Methyl bis (f,-chloroethyl) amine
hydrochloride (nitrogen mustard) gave an equivocal result. This substance has
been re-examined, and the result is included among those reported here.

In an attempt to find other initiators of carcinogenesis a further 28 substances
have been screened for this property by methods essentially similar to those
previously used. The majority were selected from the very large number of
substances which possess one or more biological activities in common with urethane,
or which are related to it chemically.

Selection of substances.

A list of substances screened is given in Table I. It was found convenient to
divide them into classes, but it is not implied that the class-names completely
describe the biological activity of each substance (e.g. nitrogen mustard is classi-
fied as an antileukaemic agent, but could equally well be classed among the sub-
stances known to exert a specific effect on cells undergoing mitosis).

* Among 45 mice which survived until the end of treatment with various doses of urethane in
conjunction with croton oil there have been 5 carcinomata and 1 sarcoma. These have appeared
6-13 months from the beginning of treatment.

12

F. J. C. ROE AND M. H. SALAMAN

The first four substances which we have examined are classed as antileukaemic
agents. These are of particular interest because of the frequent association between
inhibition of tumour growth and carcinogenesis (Haddow, 1935, 1938; Haddow
and Robinson, 1937, 1939; Haddow, Scott and Scott, 1937; Badger et al., 1942).

The antileukaemicactivity of urethane itself is well authenticated both in
human and animal leukaemia (Paterson et al., 1946; Engstrom, Kirschbaum and
Mixer, 1947; Berman and Axelrod, 1948).

1,4-Dimethanesulphonoxybutane (Myleran) was first synthesized and shown
to be an antileukaemic agent by Haddow and Timmis (1951, 1953). It is now
widely used in the treatment of human leukaemia, and is especially valuable in
the chronic myeloid variety (Galton, 1953; Petrakis et al., 1954; Boyland, 1954).
Koller (1953; 1954, personal communication) produced sarcomata in rats by the
subcutaneous injection of Myleran, and commented on the abnormal mitotic
figures in these tumours.

Triethylene melamine (T.E.M.) was synthesized simultaneously at three centres
(Haddow, 1950; Rose, Hendry and Walpole, 1950; and Burchenal et al., 1950),
and has found a place in the treatment of lymphatic leukaemia (Paterson, Kunkler
and Walpole, 1953; Bayrd et al., 1952). Rose et al. (1950) commented on the
cytotoxic effects of T.E.M., and compared them with those due to nitrogen
mustard, as described by Gaensler et al. (1948). There is evidence that T.E.M.
possesses carcinogenic activity under certain conditions. Walpole et al. (1954)
report the early development of sarcomata in rats following twice-weekly sub-
cutaneous injection of T.E.M. in arachis oil (total dosage: 0 7 to 1.1 mg./100 g.).
Shimkin (1954) reports an increased incidence of pulmonary adenomata in mice
treated with T.E.M. Hendry et al. (1951) reported an increased incidence of lung
tumours in mice injected intraperitoneally with T.E.M., but failed to induce
sarcomata in mice by repeated subcutaneous injection of the substance (0.025 mg.
weekly for over a year). Lewis and Crossley (1950) found that T.E.M. possessed
tumour-inhibitory activity against sarcomatous implants, and also reported a
decreased incidence of spontaneous lung tumours in the treated mice. Graffi,
in a review (1953) of his work and that of his colleagues, described briefly an experi-
ment in which mice were painted with T.E.M. and croton oil. No tumours resulted
from this treatment.

Nitrogen mustard has been known as an antileukaemic agent since the end
of the last war, when a leucopenia was observed in men accidentally exposed to it
(Goodman et al., 1946). Its present role in the therapy of leukaemia is reviewed
by Gellhorn (1953), Wilkinson, Haddow and Nabarro (1953), and Boyland (1954).
The carcinogenicity of nitrogen mustard has been investigated by several workers.
Boyland and Homing (1949) reported that 10 out of 14 mice injected subcutane-
ously with nitrogen mustard (1 mg./kg. weekly for 36 weeks or more) developed
tumours (8 lung adenomata, 2 lymphosarcomata, 1 uterine fibromyoma, and 1
spindle-cell sarcoma at the injection site). Griffin, Brandt and Tatum (1951)
induced similar tumours after injecting nitrogen mustard subcutaneously, intra-
peritoneally, or intravenously. Heston, Lorenz and Deringer (1953), and Shimkin
(1954), induced pulmonary adenomata in mice with nitrogen mustard. Narpozzi
(1953) tested nitrogen mustard for initiating activity on mouse skin, using croton
oil as the promoting agent. To 60 mice he gave 15 applications of 0.05 per cent
nitrogen mustard on alternate days, and after an interval of 10 days he began
applications of croton oil (5 per cent in liquid paraffin); the latter were given on

178

STUDIES ON INCOMPLETE CARCINOGENESIS

alternate days for over a year. During this period 6 mice developed up to 5
warts each, but no malignant tumours were observed. In our experience this
number of tumours may well have been due to the croton oil itself (see p. 185).
The author did not include a group of mice painted with croton oil only.

Petering (1952), Gellhorn (1953), and Boyland (1954), review the status of
aminopterin in the treatment of malignant disease, including leukaemia. Aminop-
terin has been shown to exert a specific effect on mitosis (Hughes, 1950). There
are no reports ascribing any carcinogenic action to aminopterin, or to related folic
acid analogues.

The second class of substances has been designated as exerting a specific effect
on cells undergoing mitosis.

An effect of urethane itself on the process of mitosis was reported by Ludford
(1936). He observed abnormal metaphases following treatment of tissue cultures
with 2 per cent urethane. Lasnitzki (1949) studied the effect of 0.4 per cent
urethane on growth and mitosis in cultures of normal and malignant tissues from
the mouse. The growth and mitotic rates of normal tissues were reduced to
approximately half the control levels. Cultures of C57 sarcoma and adeno-
carcinoma 63, however, showed enhancement of growth, and mitotic rates of 2
to 4 times the control values. An increased number of abnormal cell divisions
were seen in the adenocarcinoma cultures. Dustin, P. (1947b), studied the effect
of urethane on the epithelium of the intestinal crypts, and observed an effect
similar to that caused by X-irradiation. He therefore placed urethane in the
class of" radiomimetic agents" (Dustin, A. P., 1929). More recently Loveless and
Revell (1949) have pointed out that there is little evidence that urethane gives
rise to the irregular chromosome-breakage which characteristically appears 18-24
hours after exposure to X-irradiation.

The arrest of mitosis in metaphase by colchicine is well known (see Ludford
(1953) for review).

Podophyllin exerts a similar action (King, 1948; King and Sullivan, 1946).

Cornman (1947) claimed that coumarin produced metaphase-arrest in the roots
of Allium cepa and Lilium longiflorum. Ostergren (1948) found that coumarin
produced chromosome bridges and breaks in Allium cepa. Since this substance
is commonly used in some countries as a flavouring agent in tobacco (Vasic, 1953),
we thought it worth while including in the present series. It is a lactone (see p. 182).

The evidence that hydroquinone belongs to this class is dependent on the find-
ings of Zylberszac (1939), and Dustin P. (1947a), in the epithelium of the intestinal
crypts of the mouse. The latter concluded that hydroquinone exerted a radio-
mimetic action. Loveless and Revell (1949), on the other hand, doubt whether
hydroquinone should be regarded as exerting a specific effect on mitosis.

Sodium cacodylate was one of the organic arsenical compounds studied by
Dustin and his collaborators (Piton, 1929; Dustin and Piton, 1929; Dustin and
Gr6goire, 1933), and was observed to give rise to metaphase-arrest indistinguish-
able from that due to colchicine.

None of the substances included in this category can be regarded as definitely
carcinogenic for mouse skin. Harde (1939) applied colchicine to the skin of 26
mice weekly. Three mice eventually developed tumours of the skin, a further 2
mice developed tumours of other organs. Hamperl (1946) produced no tumours
in mice after painting with colchicine. Berenblum (1951) tested podophyllotoxin
on mouse skin, and concluded that it possessed slight anticarcinogenic, but no

179

F. J. C. ROE AND M. H. SALAMAN

carcinogenic or co-carcinogenic activity; Gwynn and Salaman (1953) found no
evidence of co-carcinogenic activity.

A third class of substances with narcotic properties were tested for power to
initiate carcinogenesis. They resemble urethane not only in their action on ner-
vous tissue, but also in retarding or suppressing mitosis (Ludford, 1953, for
review); and the possibility was considered that there might be some connection
between these properties and that of initiation of carcinogenesis. Urethane was
one of the narcotics used by Quastel and his associates in their study of the
chemical mechanism of hypnotic action (Quastel and Wheatley, 1933, 1934;
Michaelis and Quastel, 1941). They showed that these substances all have an
inhibiting effect on aerobic glycolysis by brain tissue in vitro. The effect is rever-
sible, i.e. when the drug is removed by washing the greater part of the glycolytic
activity reappears. Indole also has a powerful inhibitory effect on aerobic
glycolysis of brain in vitro; but in this case activity is not restored by washing
the tissue (Quastel and Wheatley, 1934).

A group of narcotics was chosen for test which included members of a wide
variety of chemical types (see Table I, Class III). Indole was also tested (Class
VI), for the reason given above.

Following the discovery by Nettleship and Henshaw (1943) of the lung tumour-
inducing property of urethane, Larsen, Rhoads and Weed (1946) examined a series
of hypnotics for the same property (including chloral hydrate, paraldehyde, and
phenobarbitone). None of the substances tested increased the incidence of
pulmonary tumours in mice.

The fourth class of substances has only two members, both of which possess a
pharmacological action in some respect similar to urethane, though they cannot be
included in the preceding classes.

o-Phenanthroline inhibits the photochemical decomposition of water by
chloroplasts in vitro, and photosynthesis in vivo (Warburg and Luttgens, 1944;
Arnon and Whatley, 1949). A similar inhibitory action has been ascribed to the
urethanes as a group (see Gaffron and Fager, 1951, for review).

Physostigmine was included because its activity as a choline-esterase inhibitor
has been attributed to the urethane group in its molecule, although urethane
itself is inactive in this respect. (Stedman and Stedman, 1931, 1932; Eadie,
1942).

The substances in the fifth class were chosen because they could be regarded as
urethane derivatives or in some way structurally related to urethane. Their struc-
tural formulae are shown in Fig. 1.

Other workers have examined urethane analogues from different standpoints.
Lefevre (1939), and Simonet and Guinochet (1939), described a colchicine-like
effect in plant cells due to treatment with ethyl N-phenylcarbamate (phenyl-
urethane). Dustin (1947b) found that ethyl N-phenylcarbamate was more toxic
than urethane, and caused similar changes in mouse intestine, whereas methyl-
carbamate was of low toxicity and caused no narcosis or mitotic disturbance.
Larsen (1947a, 1947b, 1948), studying the narcotic and lung tumour-inducing
activities of urethane analogues, concluded that methylcarbamate was inactive
in both these respects, and that increasing N-alkylation led to decreasing lung
tumour-inducing activity. He considered that N-alkylated urethanes probably
acted only after dealkylation to urethane in the body. Skipper et al. (1948) made
an exhaustive study of urethane derivatives, including methylcarbamate, ethyl-

180

STUDIES ON INCOMPLETE CARCINOGENESIS

OC2H5

/

0 =C        H

N

H

Ethyl carbamate (urethane).

OC2H5

/

0 = C       CH3

N

H

Ethyl N-methyl carbamate (Group 31).

OC2H5

/

0 = C       C6H5

N

H

Ethyl N-phenyl carbamate (Group 33).

OC2H5

/

0 =C

H

Ethyl formate (Group 30).

OC2 H6

/

0 =C

H
N

OCH3
O=C          H

N

H

Methyl carbamate (Group 30).

OC2H5

/

O    = C    C18H37

N

H

Ethyl N-octadecyl carbamate (Group 32).

SC2Hs

/

0=C          H

N

H

Ethyl thiolcarbamate (Group 34).

OC2H115

/

0 =C

CH2     H

\/

N

H

Glycine ethyl ester (Group 35).

H

Benzocaine (p-amino ethyl benzoate)

(Group 29).

FIG. 1.-Structural formulae of urethane, urethane derivatives, and related compounds.

(Group numbers refer to Table I, Class V.)

thiolcarbamate, ethyl N-methylcarbamate, and ethyl N-phenylcarbamate. The
toxicity of these four, except methylcarbamate, if expressed on a molar/body
weight basis, was greater than that of urethane. Methylcarbamate alone showed
no anaesthetic properties. Ethyl N-methyl carbamate and ethyl N-phenylcar-
bamate both caused leucopenia when administered in non-lethal doses, but neither
produced such a striking reversal of the differential leucocyte count as urethane.
Rose et al. (1950) examined some thirty simple homologues of urethane for growth
inhibitory activity against the Walker carcinoma 256 in rats. Of these only bis-
and tris-carbethoxyamine even approached urethane in activity.

181

F. J. C. ROE AND M. H. SALAMAN

Benzocaine, apart from its structural relation to urethane (Fig. 1), is of interest
in that it has a slight local anaesthetic action. Moreover it is an ester of
p-aminobenzoic acid, and thus a potential inhibitor of the action of the sulphona-
mides. A similar action has been ascribed to urethane (Johnson, Eyring, and
Kearns, 1943).

The subcutaneous injection of very large doses of ethyl N-octadecylcarbamate
in rats has been found to produce sarcomata (Walpole, 1953, personal communi-
cation). Because of this result we thought it worth while testing as an initiator.

The sixth class consists of four miscellaneous substances, 1,2-benzanthracene,
cantharidin, indole, and fl-propiolactone.

1,2-Benzanthracene was selected mainly in order to repeat the work of Graffi
et al. (1953). In their experiments 75 mice received alternate applications (each
of 3 drops) of 0-5 per cent 1,2-benzanthracene in acetone and 5 per cent croton
oil in liquid paraffin, at 3 to 4 day intervals. This treatment was continued for a
year, by which time there were 18 benign skin tumours in the 9 surviving mice.
At the same time 13 survivors of 160 mice, which had been treated with croton
oil only, showed only one tumour. Previously Berenblum (1941) recorded a
negative result from a similar experiment, in which two groups of mice were
painted weekly, one with a saturated solution of 1,2-benzanthracene in acetone,
and the other with a similar solution containing also 0.025 per cent croton oil
resin. No tumours were present in either group after 20 weeks of continuous
treatment.

Steiner and Falk (1951) reported the production of 8 sarcomata in 44 mice
injected subcutaneously with 5 mg. of 1,2-benzanthracene, and in the same paper
reviewed the findings of other workers on the carcinogenicity of this substance.
Isolated tumours of mouse epidermis, the tissue in which we are primarily inter-
ested, have been seen following skin application of 1,2-benzanthracene (Kennaway,
1930; Cook, 1933; Barry et al., 1935; and Hill et al.. 1951).

As pointed out by Steiner and Falk (1951) 1,2-benzanthracene is of interest
in the study of the relationship between carcinogenic potency and molecular
structure, because of its borderline position both in the scale of carcinogenicity
and that of physico-chemical properties (e.g. electron density at the " K " region,
photodynamic effects, and ultraviolet absorption spectrum) which have been
correlated with carcinogenicity (Mottram and Doniach, 1938; Jones, 1940;
Koffier and Markert, 1951; Badger, 1954 (review)).

The inclusion of indole has already been discussed in connection with sub-
stances of Class III (vide supra.)

CH2-CH2    w

,/-propiolactone I    I    was found to possess mutagenic activity in

0    CO i

Neurospora (Smith and Srb, 1951). Walpole et al. (1954) found it to be car-
cinogenic for the subcutaneous tissue of the rat. The latter authors commented
on the resemblance in chemical properties between this substance and ethylenei-
mine. In view of these reports, and the simple nature of the compound, we
thought it worth including in the present series of tests.

Cantharidin, a powerful skin irritant, reduced the carcinogenic effect of tar
when applied alternately with the latter to mouse skin; in this respect it resembled
mustard gas (Berenblum, 1935). Orr (1938) tested cantharidin for carcinogenic
action on mouse skin and obtained no tumours. Gwynn and Salaman (1953)

182

STUDIES ON INCOMPLETE CARCINOGENESIS

tested it for co-carcinogenic activity, with a negative result. The possibility that
this intensely toxic substance might be an initiator of carcinogenesis had thus
not been excluded. In view of the properties of f8-propiolactone it is worth
noting that cantharidin is also a lactone.

Choice of dose and mode of application.

It was not found possible to follow a uniform scheme of dosage throughout.

In the case of the chemical analogues of urethane the object was to compare
their initiating power with that of urethane itself; they were therefore used in
quantities molecularly equivalent to, or greater than, a dose of urethane the effect
of which was known. Fortunately this could be done without interference by
toxic effects. An exception was ethyl N-octadecyl carbamate, of which an equi-
molecular dose would have been difficult to apply, and which was included because
of the results of Walpole (1953, personal communication) already referred to.

In the case of nitrogen mustard, which had given a result of doubtful signifi-
cance in a previous experiment (Salaman and Roe, 1953), a dose was used which
produced most tumours in the former test.

In the other groups, whenever possible, the largest doses which just failed to
produce toxic symptoms, or other signs of general effect, e.g. narcosis, were used.
In some cases, however, the limit was set by the maximum attainable concentra-
tion in a suitable solvent, in others by the fact that strong solutions left heavy
deposits of solid substance as the solvent evaporated, which were rubbed off without
penetrating the skin.

The mode of application also varied to some extent. In the early stages of the
work it was thought that repeated doses of the initiator given alternately with
croton oil would probably produce more tumours than one, or perhaps two,
applications of the initiator followed, after an interval, by weekly applications of
croton oil. Experience has shown that initiation is either demonstrable by both
methods (e.g. urethane, T.E.M.) or not demonstrable by either (e.g. nitrogen
mustard, chloral hydrate). The methods are therefore to be regarded as quali-
tatively equivalent.

Details of dosage and methods of application are given in the following section,
and in Table I.

MATERIALS AND METHODS.

Mice.

Stock albino male mice of the " S " strain (Salaman and Gwynn, 1951; Sala-
man and Roe, 1953), fed on cubes prepared according to the Rowett Institute
formula (Thomson, 1930a, 1930b) plus fresh greenstuff twice a week, and water
ad libitum, were used throughout. They were vaccinated on the tail with sheep
lymph (kindly supplied by the Lister Institute of Preventive Medicine) as a
precaution against ectromelia. Only positive reactors were used. At the begin-
ning of experiments they were 7-9 weeks old.

Chemical substances and solvents.

Test substances.-Benzocaine (ethyl ester of p-amino benzoic acid), cantharidin,
carbromal (1-bromo-ethyl-butyryl-urea), chlorbutol (1: 1: 1-trichloro-2-methyl-2-
propanol), coumarin, ethyl carbamate (urethane), ethyl formate (ethone), ethyl

183

F. J. C. ROE AND M. H. SALAMAN

N-phenylcarbamate (phenylurethane), glycine ethyl ester hydrochloride, hydro-
quinone, indole, methyl carbamate, methyl sulphonal, ortho-phenanthroline
(AR grade), physostigmine (eserine), and sodium cacodylate, were obtained from
British Drug Houses, Ltd.

1,2-Benzanthracene, colchicine, andfl-propiolactone were obtained from Messrs.
L. Light and Co., Ltd.; chloral hydrate from T. H. Smith and Son, Ltd. (Edin-
burgh); nitrogen mustard (HN2, methyl bis (f-chloroethyl) amine-hydrochloride)
from Messrs. Merck and Co.; and phenobarbitone (luminal) from Messrs. Huffer
and Smith, Ltd. (Croydon, Surrey).

Paraldehyde (2,4,6-trimethyl-1,3,5-trioxane), and podophyllin resin (for
preparation of podophyllin extract, see below) were obtained from Messrs. Allen
and Hanburys, Ltd.

Dr. Walpole of Pharmaceutical Division, Imperial Chemical Industries, Ltd.,
kindly supplied ethyl N-methylcarbamate, ethyl N-octadecylcarbamate, ethyl
N-thiolcarbamate (thiourethane), and triethylene melamine (T.E.M., tris
ethyleneimino-s-triazine).

Mr. G. Timmis of the Chester Beatty Institute, London, kindly supplied us
with Myleran (GT 2041, 1: 4-dimethane-sulphonoxy butane).

Aminopterin (4-amino-pteroylglutamic acid) was kindly supplied to us by
Cyanamid Products, Ltd., Lederle Laboratories Division.

Croton oil.-Expressed oil of the same batch used in previous experiments in
this laboratory (e.g. Salaman and Gwynn, 1951; Salaman and Roe, 1953) was
used. It was obtained from Messrs. Boots Pure Drug Co., retailing the product of
Messrs. Stafford Allen and Sons, Ltd., 20 Wharf Rd., N. 1.

Solvents.-Acetone (AR grade), and ether (Aether Puriss. grade) were obtained
from British Drug Houses, Ltd., carbowax 300 (polyethylene glycol of average
molecular weight 300) from General Metallurgical and Chemical Co., Ltd., and
methyl alcohol from James Burrough, Ltd.

Preparation of solutions.

All solutions were prepared weight per total volume in the solvent shown
(Table I) unless otherwise stated. The preparation of glycine ethyl ester from the
hydrochloride is described in the experimental section.

The podophyllin extract used was prepared from the crude resin by extraction
with acetone. Dosage was calculated on the dry weight of the extract after
filtration and evaporation.

Technique of application.

The hair was clipped from the whole back, from forelimbs to tail, before treat-
ment and at intervals when necessary. Solutions were delivered from calibrated
pipettes, care being taken that they spread as evenly as possible over the whole
clipped area. A glass spreader was used for solutions which did not otherwise
spread easily (e.g. glycine ethyl ester hydrochloride).

Recording of tunmours.

Mice were examined throughout the course of croton oil treatment at weekly
intervals, and all tumours of 1 mm. diameter and over recorded.

184

STUDIES ON INCOMPLETE CARCINOGENESIS

The groups of mice in which tumours appeared were kept after the end of
croton oil treatment and examined at intervals for the development of malignant
tumours. Many of these mice are still under observation.

Examination of mice for lung adenomas at post mortem.

The majority of mice were killed one week after the end of the standard
course of croton oil treatment, and examined post mortem. The lungs were removed
and the surface of each lobe carefully examined, in the fresh state, for the presence
of pulmonary adenomata.

Histological examination.

Specimens of skin from mice additional to each group were removed under
ether anaesthesia three days after one and/or two applications of the test sub-
stance. Mice which died during or were killed at the end of the tests were
examined post mortem for lung adenomata and other abnormalities. Specimens
for histological examination were fixed in Zenker's fluid, embedded in paraffin
wax, and stained with haematoxylin and eosin-Biebrich scarlet (Gwynn and
Salaman, 1953).

EXPERIMENTAL.

A. Skin Tumour Production.
Treatment with croton oil alone.

A group of mice (Group 43) were reserved as croton oil controls. They received
weekly applications of 0.3 ml. 0.5 per cent croton oil in acetone. Altogether these
mice have received 72 weekly applications, but for the purposes of controlling the
present experiments only the first 18 applications need be considered, and for
convenience we have called these "a standard course of croton oil treatment."

Tumour incidence.-Table I shows that one week after the 18th application of
croton oil one of the 20 survivors bore 3 tumours. Subsequently tumours appeared
at a steady rate, reaching the levels of 1 tumour per mouse in 19 survivors after
44 applications, 2 tumours per mouse in 17 survivors after 57 applications, and 3
tumours per mouse in 12 survivors after 66 applications. So far 3 malignant
tumours have been seen in this group (after 55, 67, and 72 weekly applications
respectively).

Class I: Antileukaemic agents.

Four substances in this class were tested  nitrogen mustard, Myleran,
triethylene melamine (T.E.M.), and aminopterin. Table I gives details of treat-
ment.

Groups 1 and 2 received applications of nitrogen mustard weekly for 15 weeks
at dose-levels indicated by the results of a previously reported experiment (Sala-
man and Roe, 1953).

In a preliminary test single doses of Myleran and T.E.M. were given, which
subsequently proved to be too high (Groups 3 and 6 respectively). A majority
of the mice in both groups died, but the survivors were given the standard course of
18 weekly applications of croton oil.

185

F. J. C. ROE AND M. H. SALAMAN

0-    b

0      4.r   .
C)    I     (1

0    ..   H o .c
(1         > L 1 q

't1   0

H,

C

C
Cy

0  0  t)o 0

-4C

1-  OD  a w

2e.0 O

?40.  P

'.4 l, &O9   4..'* ,
cdP, bo I

C5

C)

0

0

DQ

0.

0

0 t

.; z

0 8

O    9!

0    %

-4

U)

00    ;;

0    4Q

Ho

4)

<4
V
?@

O 08

E-- '

? o  o  _  o  _  o  o  :  _

?  o0o  1'CO.....

- C

.* *  * . . . * .

* ~~  **

n  Do oe '. E COOCO  C

0
C)

-i   - O CO
E I .

_m to eK c

,-   .Id   *1  C

^~

') e

Ca= t^

(6 .

LC.)

0

,00

0    -4  1C O m "I

II

.)

o

U)

.0

~A-

P.)

*t!;

U)

00

W:
C.

;t

0 1 0   C O  0 1 C' a O   ,.. 0 1

:0           0

.

H s

0            O

0  1~  .O

100  ,   0

0 r-CO
- O ,- -

O C- o

*.-- 0

o  -4  er-

Cli _- "

o 00

* . .
-00

0100.

4m* m*     a * U

) >a, (D h   g z h
0  10 0    0t9~

c  cO c  oO  CO COCO

1-

o  +~  -o

*  .  *  * .* .* .

\-  e _  o1- h~

. . . .

- CO 1:

1

I * * .

0 0
. ,..?

C) Ca

O         O

0      0

V V)

.    .   .   .

o xo U:
_ -- C4

.  .   .  .

eq CO "Id U
-_ _4 _4 _

* . .
o      0 o
0i00
. . .

0 c

o .    .
C     O

* 010

010101

0   .   .

1*  * lkl

. . .

c t-O

_-  _-  _-

C r O  O0 0 O CO 0o
C A    o . .   ,

I - *..   *  ....

C   a 0 co CO CO 0

Cq

-e _ Ci _ _

----        - S_~_

0 O o0 CO 00o1o0_
* 10

U) 010100001
U)   -        -

00000000

0 ^ 10 CDXO 010
CO 011 Ci   0 -0

01   CO

-4

00

o

*  -.  *  . .  . .

0a 0

0 0 - 0 1  q C c 4   1 0 0
- 0 1 0 1 0 1 0 1 0 10e q

186

I.~

*CO>

e,j

I-I

*C4

pq
E-

L-9

'"

STUDIES ON INCOMPLETE CARCINOGENESIS

o       C~~~~~o

0,~ ,

E                ?
H                      O

0 ~0
CD 0

- ; CQ

1  4a

0Q4a

-P

L 0 O

n:0

P. 4.

Cao

0

C)

w
Q

-i2

B

(D

0)
-P

s

.9

-P.'

0

0
(D

oN      O'      .     I'-  0 o

0q-4-        --O     o-'    O     -

00            0 0  0       0     ~

cs _ _ _ es ~~~~~~~~~~~~~

*  *      .  o

Or.-      o' ~,~

-        - -
00        00

++

0

,, C >

.,?

0 0

;)  0

* .2
ce *t

o

o > o~

-
so 0 0

-P-W

O

0 _

0 CC

Xoo}

H    -P.-o

COe

0

f4

0

z

c.L

0

I-l

r0

r-       O      '

,-    G"N  ,--I

- 0           -

N       0     CO

* *         *    W

;,-a,r          0

C  mCO  CO  C    C

o \.

so
COm

00

- - 0

'10

?  o?

04_

0

c  0 o0  a3 o    o  r  O

,O   O  , ,O  N   0

,C O  ,-~ [-- O O C  c,- c o
- -    -    - CO..-  -.

-   0  -    N 0 0  4   041r-

*

IC

O          coz

a)

0

Op "

C3

CO*    COO  N    * * ,   *

14 4

.1   (D =  -    ( h

o^
o
0

4 ^

.    .   .   . .

N,        CC  _-

C   -

*  .   ,*

F4              C'

~~~~~~~~ce

4-P   :4-  ;14 C3   O4 C3 0 O

O t   C   ;   1 0

? 0 4      C O ? -   -

?C
? D'

0

0

CO

0

0

lo

0

(S)

(1) -4-D

.5

Ca

Q   r-4
0

N

9 --q

(3) >?-
pq -.0

rA

oo   oo

0 0  0,  0 C"

0 4 0   . * 0 4

:0     o     o

-      0 4    0c

0

CO

(00

-.

N

10

0

-4

0

._4

Ca

CC
._q

,--

04

h 4

a )

Cli

_- Io,

,. a

.   .   .   .

oooCO

....          00

CO C U I

-   -   -  1_ 0

-q0

*   .   .   .

0

-4-D~~~~~

,, , .  .  * .  .  .4,
oo  o  o  000
(3oO  O ~  OOO CCO

*  P  . . .. *.  .

CO  0  0  0 0 0 0(2   0 0

-   0 4   0 4   0 4 ~ ~ ~ ~ ~ 0 4   04 0~4

Nr oo    c o 0  -  04  CO          L 10  C-      N C O O    -- c

040c  e  =    c(  O  cO  c      CO  to   co      COCOC 4 c  s P  +

COi

0

CO    t

*4-
0

0

CD

.)v

o . 0
*     w

.o
-  0

0

0

.o
S o  0
, .  -i

0
0

o   o

0

o    0

00

Oa

?0

0

oo

~0

00

~0

-o    o  o

O~~C

o3. -i-.' +-

M ,
9

o. o X

0 -P

0

.     o
O    0

*   -1--  4-

C)4O .

ou

? +

187

F. J. C. ROE AND M. H. SALAMAN

Groups 4 and 5 were painted weekly with Myleran for 10 weeks, receiving alto-
gether a higher total dose than mice in Group 3. As in all groups in which the
test substance was given in divided doses at weekly intervals the first applications
of croton oil alternated at 3 to 4 day intervals with those of the test substance.

Group 7 was given a single application of T.E.M., and Group 8 weekly applica-
tions of T.E.M. for 15 weeks. In both groups croton oil treatment was begun
3 days after the first application of T.E.M. When the standard 18 applications
had been given it was decided to continue them for a few weeks, since at that time
the number of tumours was increasing rapidly.

Groups 9 and 10 were given T.E.M. treatment corresponding to that of Groups
7 and 8 respectively, but no croton oil treatment thereafter.

Considerable difficulty was encountered in preliminary solubility and toxicity
tests with aminopterin. The most suitable solvent for skin application was
found to be a saturated solution of sodium bicarbonate in methyl alcohol (approxi-
mately 0.05 per cent). This solution was made up immediately before use.
A single dose of 0.3 ml. 0.02 per cent aminopterin in this solvent was given to 20
mice in Group 11. This dose proved to be too high, and many died. Subsequently
the survivors, together with 5 hitherto untreated mice, received 5 weekly applica-
tions of 0.3 ml. 0-01 per cent aminopterin in the same solvent. All mice began
a standard course of croton oil treatment 3 days after the first application of
aminopterin.

Tumour incidence.-The number of tumour-bearing mice, and the incidence
of tumours in mice surviving until one week after the end of croton oil treatment,
are shown in Table I. One week after the 18th application of croton oil 47 tumours
were present in 27 survivors (Groups 6, 7, and 8) which had received treatment with
T.E.M. and croton oil. This tumour incidence increased with further croton oil
treatment. Fig. 2 shows the rates of development of tumours in these groups and,
for comparison, that in Group 43 which received croton oil treatment only.

A significance test for the difference between the mean numbers of tumours
per mouse in Groups 6 and 7 (pooled) and in Group 43, gives t = 2.90 on 35
degrees of freedom, a value which could be exceeded by chance with a probability
of P = 0.006. The comparison between the mean numbers of tumours per mouse
in Group 8 and in Group 43 gives t = 2-42 on 28 d.f.; P = 0.02. No tumours
were observed in 17 survivors which received T.E.M. only (Groups 9 and 10).

It is concluded that T.E.M. is an effective initiator of skin carcinogenesis in
the mouse, but not carcinogenic in the doses used.

Nitrogen mustard and croton oil (Groups 1 and 2) produced 5 tumours in 19
survivors at the end of croton oil treatment. This was in accordance with our
previously reported borderline result with this substance (Salaman and Roe,
1953), the mean number of tumours being slightly greater than that obtained with
croton oil only, but not significantly so (t = 0-57 on 37 d.f.; P = 0.57).

The distributions of the number of tumours on individual mice, in all the groups
reported in this paper, are markedly skew. The significance levels indicated by
the t-tests will be somewhat affected by this fact. Moreover it is possible that
small differences in the incidence of tumours will be more readily detected by some
quantity other than the mean number of tumours per mouse. For the comparison
of Groups 1 and 2 (pooled) with Group 43, for instance, it is possible to rank
together the mice in both series in order of number of tumours borne, and to ask
whether the mean rank for Groups 1 and 2 (pooled) differs significantly from that

188

STUDIES ON INCOMPLETE CARCINOGENESIS

for Group 43. The appropriate test (Wilcoxon's) is described by Kruskal and
Wallis (1952, pp. 590-592). Formulae for the expected value and variance of R,
the mean rank for mice in, say, the control group (it is immaterial which group is
chosen), are given by Kruskal and Wallis. The square of the difference between R
and its expected value, divided by its variance, is distributed approximately as X2
on 1 d.f. Application of this test to the series in question gives X2 = 1-90 on 1 d.f.;
P = 0-16. The difference is still not significant. However in some other groups,
as shown below, the ranking test indicates a significant difference at the customary
5 per cent level, whereas the t-test does not.

ao

r.
.-4

m        I

W m

bt-

?)

Time in weeks from beginning of croton oil treatment.

FIG. 2.-Initiating effect of triethylene melamine (T.E.M.).

A - -  -A Groups 6 and 7 (combined): 0-5 mg., and 0-24 mg. T.E.M. respectively

(given as a single application in each case) followed by 20 weekly appli-
cations of of 0-5 per cent croton oil (beginning 3 weeks after treatment
with T.E.M.).

O          0 Group 8: 1-8 mg. T.E.M. (15 weekly applications of 0.04 per cent) and

22 weekly applications of 0.5 per cent croton oil, alternating at first with
the former.

x -.-------- x Group 43: Control group: weekly croton oil treatment only.

Acetone was the solvent throughout. The numbers of mice in each group at the beginning
of the experiment and further details of treatment are shown in Table I. Numbers of
survivors are shown in brackets.

Combinations of Myleran and croton oil (Groups 3 to 5) produced no more
tumours than croton oil alone.

An attempt was made to give a higher total dose of Myleran. As in all experi-
ments involving skin applications it is difficult to say how much of the apparent
toxic effect of a substance is due to absorption from the gastro-intestinal tract
following licking, and how much to absorption through the skin. To ascertain
these proportions in the case of Myleran a group of 10 mice were prevented from
licking themselves by means of plaster of Paris collars; 5 were then painted with
10 mg. Myleran to the clipped dorsal skin, and the remaining 5 mice similarly
with 5 mg. Thereafter mice were kept in separate compartments to prevent their
licking one another. The collars were removed after one week. All the mice
which received 10 mg. Myleran died within 3 weeks, but those which received
5 mg. remained healthy. The latter were again put in plaster collars and given a

189

I

F. J. C. ROE AND M. H. SALAMAN

further application of 5 mg. each. Within 3 weeks of this second treatment all
5 were very sick or dead.

It was concluded that Myleran in the maximum tolerated dose was not an
initiator of carcinogenesis in mouse skin, and that toleration could not be increased
by the prevention of licking.

Mice treated with aminopterin and croton oil (Group 11) developed no tumours.

Class II: Substances exerting a specific effect on mitosis.

Five substances in this class were tested: colchicine, coumarin, hydroquinone,
podophyllin, and sodium cacodylate. Details of treatment are given in Table I.

Tumour incidence.-In the doses employed none of these substances showed
any evidence of initiating activity.

Class III: Narcotic agents.

Six substances were included in this class: carbromal, chloral hydrate,
chlorbutol, methyl sulphonal, paraldehyde, and phenobarbitone. Details of
treatment are given in Table I.

Tumour incidence.-In Group 20, which received two applications of 4 per cent
chloral hydrate with a week's interval between them, followed by a standard
course of croton oil, 4 tumours were present on 17 survivors one week after the end
of croton oil treatment. The mean number of tumours does not differ significantly
from that in Group 43 treated with croton oil only (t- 0-93 on 35 d.f.; P-
0.36). The ranking test gives X2 = 2.30 on 1 d.f.; P = 0.13. The substance
was re-tested at a higher dose (Group 21: 15 weekly applications of 5 per cent).
However in this group also the yield of tumours (4 in the 20 survivors) did not
differ significantly from that in mice receiving croton oil only (Group 43).

In Group 25 which received 2 applications of 2 per cent phenobarbitone
with an interval of one week between them, followed by a standard course
of croton oil treatment, only one papilloma was present a week after the final
application of croton oil. However a week later an ulcerated tumour appeared
which was found, on histological examination, to be a fairly well differentiated
squamous carcinoma which had penetrated the panniculus. Although an isolated
finding this was, in our view, a remarkable one. We have never seen a malignant
skin tumour arising spontaneously in mice of the strain used for these experiments.
However, as described above, in Group 43, which received croton oil weekly for
up to 72 weeks, 3 carcinomata have appeared, though none before the 55th week.

Because of this isolated finding phenobarbitone was re-tested at a higher dose
level, in Group 26. However, no malignant tumours appeared in the group. It
may be profitable to explore this part of the field again when we have learnt
more of the process of initiation, and of the development of malignancy.

Class IV: Pharmacological analogues of urethane not included in Classes I, II

and III.

Two substances in this class were tested: o-phenanthroline and physostigmine.
Details of treatment are given in Table I.

Tumour incidence.-No tumours were seen in either group at the end of the
standard course of croton oil treatment.

190

STUDIES ON INCOMPLETE CARCINOGENESIS

Class V: Urethane derivatives and related compounds.

Eight substances were included in this class: benzocaine (p-amino ethyl
benzoate), ethyl formate, ethyl N-methyl carbamate, ethyl N-octadecyl carba-
mate, ethyl N-phenylcarbamate, ethyl thiolcarbamate, glycine ethyl ester, and
methyl carbamate. Details of treatment are given in Table I.

The solution of glycine ethyl ester (Group 35) was made freshly each week
immediately before use, by the following method. The solvent was first prepared
by adding 1 volume of carbowax 300 to 4 volumes of distilled water. A 30 per
cent w/v solution of glycine ethyl ester hydrochloride was then made up in this
solvent. To this solution aqueous sodium hydroxide was added drop-wise until
it was neutral to phenol red. Finally the volume was adjusted, by adding
more of the prepared solvent, to make a 19 per cent w/v solution of glycine ethyl
ester.

Tumour incidence.-Of 17 survivors which received 250 mg. ethyl N-methyl
carbamate followed by a standard course of croton oil (Group 31) 7 bore a total
of 9 tumours at the end of treatment. The difference between the mean number
of tumours in Group 31 and that in Group 43, is not significant by the t-test
(t - 1.57 on 35 d.f.; P  0.12). The ranking test, however, gives X2 = 6.2
on 1 d.f.; P - 0.013. In Group 33, which received 500 mg. of ethyl N-phenyl
carbamate, of 19 mice surviving until the end of croton oil treatment 6 bore a total
of 16 tumours. The difference between the mean number of tumours in Group 33
and that in Group 43 does not quite reach the customary level of significance by
the t-test (t  1*87 on 37 d.f.; P = 0.07), but the ranking test givesX2 _ 4.3 on
1 d.f.; P = 0.04. It must be emphasized that the ranking test was chosen after
the data had been examined, and perhaps exaggerates the significance.

Fig. 3 shows the rates of development of tumours in Groups 31, 33, and 43,
together with that in a group from a previous experiment (Salaman and Roe,
1953) which received a dose of urethane (240 mg.) approximately equivalent on a
molar basis to those of the test substances in Groups 31 and 33.

We conclude that ethyl N-methyl carbamate and ethyl N-phenyl carbamate
probably both possess some initiating activity, but of a lesser degree than that of
urethane itself. Two further groups of mice are at present under test, both receiv-
ing ethyl N-phenyl carbamate at higher dosage than Group 33. One of these
groups will receive no croton oil treatment, in order to determine whether the test
substance is itself carcinogenic for mouse skin. (See Addendum II).

Ethyl thiolcarbamate (Group 34), given as three applications of 20 per cent
w/v in acetone at intervals of 4 days, followed by croton oil treatment, gave rise
to a solitary tumour in the 16 surviving mice. In a previously reported experi-
ment (Roe and Salaman, 1954) a group of mice received three applications of
20 per cent w/v urethane in acetone at intervals of 4 days, followed by croton oil
treatment; in this group all of the 18 survivors bore tumours, and the total
tumour yield was 70. We conclude that the substitution of a sulphur atom for
the ester-linkage oxygen atom deprives the urethane molecule of its initiating
activity.

Benzocaine, ethyl formate, glycine ethyl ester, and methyl carbamate, all
applied in relatively high doses, showed no evidence of initiating activity.

Ethyl N-octadecyl carbamate in the dose given (see introduction) was without
initiating effect.

191

m1   P-   ( -

ax O-

0.

4.0

0

3-0
to

? 20
0 ,0

1 -0
0

0 3Q

F. J. C. ROE AND M. H. SALAMAN

/+(22)

/

/
/

,,+-.-+/

/
/

- ~~~/

.J

-         /               ~ o('19)'

I       I   ._x-.-.(20) I

-     6      8     10    12    14    16    18     20

Time in weeks from beginning of croton oil treatment.

FIG. 3.-Initiating effect of urethane derivatives compared with that of urethane itself.

~A       A/\ Group 31: 250 mg. ethyl N-methyl carbamate (2 applications of 42 per

cent with an interval of one week) followed by a standard course of
croton oil treatment (18 applications of 0.5 per cent).

O-- - -0 Group 33: 500 mg. ethyl N-phenyl carbamate (2 applications 13.9 per cent

on 1st, 3rd, 5th, 8th, 10th, and 12th days) followed by a standard
course of croton oil treatment.

t-  -+ A group from a previous experiment: 240 mg. urethane (2 applications

20 per cent w/v with an interval of 15 minutes, on 1st and 8th days)
followed, 4 weeks after the 1st application of urethane, by a standard
course of croton oil treatment.

x- -------- x Group 43: Control group: weekly croton oil treatment only.

Acetone was the solvent throughout. The numbers of mice in Groups 31, 33, and 43 at
the beginning of the experiment are shown in Table I. The group receiving urethane and
croton oil consisted of 26 mice. Numbers of survivors at the end of the experiment are
shown in brackets.

Class VI: Miscellaneous substances.

The four substances included in this class were 1,2-benzanthracene, cantharidin,
indole, and f,-propiolactone. Details of treatment are given in Table I. In the
case of Group 37, painted with 1,2-benzanthracene and croton oil, treatment with
the latter was continued for two weeks beyond the standard course, since at that
time the number of tumours was increasing rapidly.

Tumour incidence.-In mice which received 1,2-benzanthracene and croton
oil (Group 37), one week after the 18th application of the latter there were 7
tumour-bearing mice and a total of 21 tumours among the 18 survivors. At this
stage the mean number of tumours differed significantly from that in Group 43
(t = 2.29 on 36 d.f.; P- 0-03). When two further applications of croton
oil were given the incidence rose to 43 tumours in 13 mice out of 18 survivors.
The rate of development of tumours is shown in Fig. 4. A control group which
received similar doses of 1,2-benzanthracene without croton oil treatment devel-
oped no tumours (Group 38). Moreover no tumours appeared in a further group
which were painted weekly with 1 per cent 1,2-benzanthracene for 15 weeks

192

STUDIES ON INCOMPLETE CARCINOGENESIS

193

(Group 39). We conclude that 1,2-benzanthracene is an initiator of carcino-
genesis for mouse skin, but not carcinogenic in the doses given.

Of the 20 mice receiving applications of cantharidin together with a standard
course of croton oil (Group 40) 17 survived until the end of treatment, and of these
4 bore a total of 6 tumours. The mean number of tumours does not differ signifi-
cantly from that in Group 43 either by the t-test (t = 0-97 on 37 d.f.; P =  0.34),
or by the ranking test (x2 - 2-45 on 1 d.f.; P = 0.12).

Indole (Group 41) showed no evidence of initiating activity.

0

m 6
~0

? ~ 5.

,~4.

a)

o

.e.  5.1
.q

Z

; 42
1)

.0

0CZ
(D

4

4D
a

?)

Time in weeks from beginning of croton oil treatment.

FIG. 4.-The initiating effect of 1,2-benzanthracene compared with that of

9,10-dimethyl-1,2-benzanthracene (DMBA).

*          *. Group 37: 6 mg. 1,2-benzanthracene (2 applications of 1 per cent with an

interval of one week) followed by a standard course of croton oil treat-
ment (18 applications of 0.5 per cent).

O     -    C A group from a previous experiment: 0 3 mg. DMBA (single application

0'2 ml. 0*15 per cent) followed, after an interval of 4 weeks, by a
standard course of croton oil treatment.

x ----- x Group 43: Control group: weekly croton oil treatment only.

Acetone was the solvent throughout. There were 20 mice in each group at the beginning
of the experiment. Numbers of survivors are shown in brackets.

After the first application of 10 per cent 8-propiolactone mice of Group 42
showed considerable ulceration and scabbing of the skin. The beginning of croton
oil treatment was therefore postponed, and the concentration of -propiolactone in
the second application reduced to 5 per cent. However the inflammatory skin
reaction persisted, and all treatment was withheld for a further fortnight. By then
the skin lesions had healed, and weekly applications of 2-5 per cent ,8-propiolactone
were begun.    The skin reaction to this concentration was minimal.        Weekly
croton oil applications were begun 3 days later. Of 19 survivors one week after
the final application of croton oil 15 bore a total of 160 tumours. The mean
number of tumours differs significantly from that in Group 43 (t = 3.55 on 37 d.f.;
P = 0.001).   The rate of development of tumours is shown in Fig. 5. In 3 mice

13

PF. J. C. ROE AND M. H. SALAMAN

tumours appeared before the 6th application of croton oil, which was earlier than
tumours normally appear in mice painted weekly with croton oil following treat-
ment with an initiator. We are therefore led to suspect either that,8fl-propiolactone
is carcinogenic for mouse skin, or that the scarring resulting from the first two
applications of f,-propiolactone acted as an additional promoter of tumour forma-
tion. The substance is being tested again, both by itself and together with croton

n-A

U *s

8.0

0

bO  7 0

6 0

Go

-0

$:  51]

0

4-0

0

;, 31]
0

~0

1-0
i   2-0
0

0    1 1

(19)

-

k       _           I    I   I    L_-z--(2

0     2    4    6    8    10   12   14   16   18
Time in weeks from beginning of croton oil applications.

FIG. 5.-Tumour incidence in mice treated with fi-propiolactone and croton oil.

o          0 Group 42: 155 mg. ,l-propiolactone (14 weekly applications, beginning

at 10 per cent and falling to 2-5 per cent, see text) and 18 weekly appli-
cations of 0 5 per cent croton oil alternating at first with the former.
x -------- x Group 43: Control group: weekly croton oil treatment only.

Acetone was the solvent throughout. Both groups consisted of 20 mice at the beginning
of the experiment. Numbers of survivors are shown in brackets.

Note.-In Group 42 tumours began to appear after only one week of croton oil treatment
(i.e. 6 weeks earlier than in any other group).

oil. There is no doubt that ,8-propiolactone possesses initiating activity, but it
is not yet possible to say whether in non-ulcerating doses it has promoting power
as well. (See Addendum I.)

B. Histological Findings in the Skin.

The presence or absence of hyperplasia 3 days after the first and/or second
applications of the test substances is shown in Table II.

Of the substances which, in conjunction with croton oil, gave rise to skin
tumours, T.E.M., ethyl N-methyl carbamate, and ethyl N-phenyl carbamate,
caused no hyperplasia of the mouse epidermis when applied alone. The reaction
to fl-propiolactone varied remarkably from mouse to mouse: one mouse three days

194

STUDIES ON INCOMPLETE CARCINOGENESIS

TABLE II.-Histological Appearances in Mouse Skin after One or Two

Weekly Applications of the Test Substances.

Substance.
Nitrogen mustard
Myleran   .
T.E.M.

Aminopterin

Colchicine .
Coumarin .

Hydroquinone
Podophyllin

Sodium cacodylate

Carbromal .

Chloral hydrate
Chlorbutol .

Methyl sulphonal
Paraldehyde

Phenobarbitone

o-Phenanthroline
Physostigmine
Benzocaine

Ethyl formate

,, N-methyl carbamate

,, N-octadecyl carbamate

,, N-phenyl carbamate

thiolcarbamate .
Glycine ethyl ester

Methyl carbamate
1,2-benzanthracene
Cantharidin
Indole    .

,f-propiolactone
Croton oil .

Concentration

in acetone
(per cent).

0.1

0-67
0-17
0-02

(in saturated solution of

NaHCO3 in methyl

alcohol)

0-33
15-0
6-7
0.1
28-0

(in methyl alcohol)

5.0
4.0
16-0
8.0
50-0
2-0
10.0

0-05
27-0
100-0
42-0

6-7

(in ether)

20-0
20-0
19.0

(in 20 per cent aqueous

carbowax 300)

25-0

1.0
0.01
2-0
2-5
0-5

after the 2nd application had an apparently normal epidermis, whilst others
similarly treated showed quite marked hyperplasia. 1,2-Benzanthracene con-
sistently gave rise to a moderate epidermal hyperplasia.

Of the substances which gave borderline results in the tests for initiation,
cantharidin caused moderate epidermal hyperplasia. The reaction of the skin
to nitrogen mustard has been described in a previous report (Salaman and Roe,
1953).

Both colchicine and podophyllin gave rise to striking histological appearances
in mouse skin. In both cases the epidermis was thickened and many of the cells
were greatly enlarged. Many clumped metaphases were seen, as well as aberrant
chromosomes, fragmentation of chromatin, multinucleate cells, and a considerable
number of pyknotic nuclei. These appearances have been fully described by other
authors (King, 1948; King and Sullivan, 1946).

Day of
biopsy.

3rd and 10th

3rd

3rd and 10th

3rd

10th

3rd and 10th

3rd

3rd and 10th

3rd

3rd and 10th

3rd

3rd and 10th

10th

3rd and 10th

3rdJ 9   13

Epidermal

hyperplasia.

++

0
0
0

0
0

+++

0
0

0
0
0
0

++
0.
0
0
0
0
0
0

0
0

+
0
0
0
0
0

0

++
++

0

Oto ++
+ + +

195

F. J. C. ROE AND M. H. SALAMAN

c. Induction of Lung-adenomata.

The experiments were not designed for the study of the induction of lung
adenomata. Skin application would not have been the method of choice for that
purpose. However, it is worth recording that the incidence of lung adenomata,
as seen at post mortem one week after the end of croton oil treatment, was much
greater in a few of the groups than in the rest.

Ethyl N-methyl carbamate followed by weekly croton oil gave rise to 26
adenomas in 9 mice out of 17 survivors, a result which confirms that of Larsen
(1948) obtained by intraperitoneal injection of the substance alone.  Methyl
carbamate and croton oil gave rise to 12 adenomata in 7 mice out of 18 survivors;
Larsen (1947b) failed to induce adenomata by intraperitoneal injection of this
substance alone.

Ethyl formate and croton oil gave rise to 15 adenomata in 6 mice out of 13
survivors; the induction of lung adenomata by this substance has not been
previously reported.

Ten mice treated with T.E.M. and croton oil (Groups 6, 7, and 8) have so far
been examined post mortem at periods of 22 to 35 weeks after the end of croton oil
treatment; the remainder are still under observation for the appearance of malig-
nant skin tumours. Seven of the former bore a total of 63 lung adenomata.
Although this result is not strictly comparable with those in the other groups,
it suggests that treatment with T.E.M. followed by weekly croton oil produces
a significant number of adenomata.

Of the mice painted with croton oil weekly for up to 72 weeks (i.e. 4 times the
duration of croton oil treatment received by other groups), some of which are
still under observation, 15 mice have so far been examined post mortem. Ten of
these bore no adenomata, the remaining 5 bore 20, 3, 2, 1, and 1 respectively. It
therefore appears that croton oil is capable of producing some lung adenomata,
but at present the evidence depends mainly on the findings in a single mouse. It
should be emphasised that this result cannot be used as a control for other groups
because of the much longer period of croton oil treatment and greater age of the
mice at the time of examination in this group.

Among the other groups still under observation for the development of malig-
nancy (9, 10, 37, 38, 39, and 42) data on the incidence of lung adenomata is at
present inadequate.

In all the remaining groups the mice were killed one week after the end of
croton oil treatment. The total incidence was 45 adenomata in 394 mice examined,
and did not exceed 1 adenoma per 4 survivors in any one group.

These results cannot be satisfactorily assessed until the rest of the mice still
under observation have been examined post mortem, and until the incidence of
lung adenomata in mice treated with the standard course of croton oil alone is
known.

DISCUSSION.

As a sequel to the finding that urethane acts as an initiator of carcinogenesis
for mouse skin (Salaman and Roe, 1953) the results of the screening of 29
substances for similar activity have been recorded. Many of these substances
were selected for test because they possessed some other biological activity in

196

STUDIES ON INCOMPLETE CARCINOGENESIS

common with urethane, or could be regarded as structurally related to it. The
reasons for selecting each particular substance are given in the introduction,
together with a brief survey of the findings of other authors.

Three of the 29 substances tested in conjunction with croton oil by the methods
described gave definite evidence of initiating activity, namely T.E.M., 1,2-ben-
zanthracene, and 8-propiolactone. Two of the derivatives of urethane tested,
ethyl N-methyl carbamate and ethyl N-phenyl carbamate, each produced several
tumours, but the differences between the mean number of tumours in either of
these groups and that in the group treated with croton oil alone did not
reach significance at the customary 5 per cent level when judged by the t-test.
By a ranking test (Kruskal and Wallis, 1952),however, these differences were
judged significant. The reason for using the latter test and the need for caution
in accepting the results of its application have been given above. Three other
substances, nitrogen mustard, chloral hydrate, and cantharidin, gave rise to a
few more tumours than were seen in mice treated with croton oil alone, but the
differences were not statistically significant by either the t-test or the ranking
test. The remaining substances, which included Myleran, aminopterin, colchicine,
podophyllin, and six narcotic agents, showed no evidence of initiating activity.

The base line against which initiating activity was measured was the tumour
incidence in mice treated with croton oil only, for the same period as the test mice.
Three papillomas developed in a group of 20 croton oil treated mice during this
period (i.e. 18 weeks). But when applications were continued the incidence of
tumours steadily increased until, at 72 weeks, an average of over three tumours
per surviving mouse was reached. Moreover three malignant tumours appeared
between the 13th and 17th months of treatment. Although precautions against
contamination of the test mice with traces of carcinogenic hydrocarbons, or other
initiators, from cages or fomites, or in the process of handling, were always taken,
the possibility of such contamination cannot be excluded. Alternatively, it may
be that croton oil, besides being the most powerful known promoting agent for
mouse skin, has also initiating power of a low order. It must be remembered,
however, that spontaneous skin tumours do occur in mice (Slye, Holmes and
Wells, 1921) although we have not encountered them in the " S " strain used for
these experiments; and it is possible that latent tumour foci are present in normal
mouse skin, which do not develop into tumours in the life-time of untreated mice,
but may be induced to do so by prolonged treatment with croton oil.

The incidence of malignant tumours of the skin in mice treated with initiators
and croton oil, and in mice treated with croton oil alone, is under study at present.

In the five groups in which initiating action is considered to have been demon-
strated, not only did tumour incidence at the end of croton oil treatment exceed
that of the croton oil controls, but tumours appeared much earlier. Perhaps the
most interesting findings is the initiating action of T.E.M. If total dosage is
calculated on either a weight or a molar basis T.E.M. is far more effective than
urethane. It is also far more toxic. Like urethane it does not give rise to skin
tumours when applied repeatedly in maximum sublethal doses without croton
oil, nor to any epidermal hyperplasia or other recognisable histological change.
Moreover, like urethane it possesses antileukaemic, antimitotic, and lung adenoma-
inducing action. The only striking differences in biological activity between the
two substances are the hypnotic action of uretha ne, and the production of
subcutaneous sarcomata by T.E.M. (Walpole et at., 1954).

197

F. J. C. ROE AND M. H. SALAMAN

The findings in the case of Myleran are of interest since, though an anti-
leukaemic agent, and a carcinogen in rats, it failed to initiate skin tumour
formation or to produce lung adenomas in the mouse. In the last respect our
findings confirm those of Shimkin (1954). In view of these negative results it
seems desirable to confirm the carcinogenicity of this substance for the subcutis
of the rat (Koller, 1953; 1954, personal communication).

Cornman (1954), discussing the activity of a series of carbamates, concludes that
"almost any radical added to urethane increases its effectiveness as a mitotic
poison and decreases or removes its carcinogenic action" (i.e. lung adenoma
induction). Our results suggest that almost any modification of the urethane
molecule is likely to decrease or remove the initiating activity for mouse skin.
Moreover, none of the antimitotic substances in Class II (see Table I) of the present
paper showed evidence of initiating activity.

Our findings in respect of lung adenoma induction by urethane derivatives
agree in the main with those of Larsen (1947a, 1947b, 1948) who tested a wide
range of these substances. However the rather high incidence in the group
treated with methyl carbamate and croton oil is at variance with his findings.
The fairly high incidence in mice treated with ethyl formate and croton oil is also
of interest. Both these results require confirmation.

Among the test substances unrelated to urethane two showed initiating action.
The finding that 1,2-benzanthracene is an initiator confirms the result of Graffi
(1953). In view of the relatively small dose (6 mg.) required to initiate skin
tumour formation it is possible that the low carcinogenicity of this substance
when applied alone (see introduction) is due to its relative lack of promoting
activity. This result suggests that it would be worthwhile testing other sub-
stances on the borderline of carcinogenicity for initiating action.

,f-propiolactone is undoubtedly an initiator of carcinogenesis in mouse skin.
However the fact that tumours appeared as early as the second week of croton oil
treatment suggests that it may prove to be a carcinogen for mnouse skin, as it is
for the subcutaneous tissue of the rat (Walpole et al., 1954). A further suggestion
that this is so is given by the fact that 4 malignant tumours have already appeared
among 19 surviving mice only 25 weeks after the beginning of treatment with
fi-propiolactone and croton oil. Malignant tumours have only appeared in other
groups much later than this. It will not be possible to decide upon the status
of this substance until further tests, now under way, are complete. (See
Addendum I).

Table III shows the relation between the various biological activities (as far
as they are known) of a number of the substances tested. There are many gaps,
and some of the negative findings refer to experiments too limited to exclude
a weak positive action. However it is perhaps permissible to suggest that there is
some correlation between tumour-initiating activity for mouse skin, adenoma-
inducing activity for mouse lung, and carcinogenicity for other species or tissues.
But there seems to be no correlation between initiating activity and antimitotic
or hypnotic activities, or between initiating activity and the ability to cause hyper-
plastic changes in the epidermis.

It is perhaps not surprising that substances which are capable of inducing
tumours of the lung or other tissues in the mouse, or in other animals, act as
initiators of carcinogenesis in mouse skin. Indeed the likelihood of this was the
reason for selecting several of the substances. However the range of substances

198

STUDIES ON INCOMPLETE CARCINOGENESIS

0             0

34  Ca

p~

o .  4 o  oo  o - I-)

00

0o    o  + 0

0- Q oo   +

>_ Ca  oot

I:L

+

v4

C) 4

.~  .  .  . .

C)++++

.;   + + + + +0

k  . *  *

0     0

Ca

Q4  b O

2 3.'

o  o   o

-

? . . ? . ?

4-4 ~o ~ ++  +

H   .0

~044 , +
-   4 4

+

+
+

0

-4

o

0

+-

+0o

+-

o  o  o o  ;X

o  0   00

34

0

.   . .

Ca

-4

0

++

0

4
* * .  .  4a

Ca

U,

++     0

C)
I,
, 10

34

0

0
C)

341

k

0

34

C1)
.3

4

*  .   - e  :

00 C)

OC)

*   *  34

I

+     @

+ + 00 ?,

+     C)
T      0

0

*         *   ;21

C1)

0

0

0
._~

34
04

P4
0o

CD
. 4

C) -

=0

V

199

344

0

0

._q
44
C)

OQ

._

344
0
3-4
0

o
C)

0
f-i

34

44
O

O
0

Q
._

4-

C)

0
0

-44

U,
b
341.
C)

co

._

C1)
0

44

44

la

34-

0
a)
k

44

f0
3)
C)

C)

z4

b~.w

o0b

0

._

4 4

a0

-Y C)

O 44

0 rt
C)

C)

*_

1:1

0
D

4
Q

0)

0

* C4
ez

4Z

2
zct

o~-
* IO
.4

.4

)

0

* 44b

1.

H:

F. J. C. ROE AND M. H. SALAMAN

so far tested is small, and the possibility is not excluded that initiators may be
found which by themselves do not give rise to tumours in any tissue. Certainly
the fact is established, and has, we think, practical as well as theoretical import-
ance, that there exist substances which under certain conditions produce no
tumours, nor any other recognised change, in a tissue, yet alter it in such a way
that when a different stimulus is applied, perhaps much later (Roe and Salaman,
1954), benign and malignant tumours appear.

SUMMARY.

1. As a sequel to the demonstration that urethane applied to mouse skin
followed by repeated applications of a promoting agent (croton oil) acts as an
initiator of carcinogenesis, 29 substances, most of them related pharmacologically
or chemically to urethane, have been screened for similar activity.

2. Of four antileukaemic agents tested, triethylene melamine (T.E.M.) was
found to be an effective initiator of carcinogenesis but not carcinogenic, for mouse
skin, in the doses tested; nitrogen mustard, Myleran, and aminopterin, in maxi-
mum sublethal doses, showed no initiating activity.

3. Of five substances exerting a specific effect on mitosis (in addition to those
included in the category of anti-leukaemic agents), none showed initiating activity.

4. Of six narcotic agents tested none showed unequivocal evidence of initiating
activity.

5. Of eight urethane derivatives and related compounds, ethyl N-methyl car-
bamate and ethyl N-phenyl carbamate showed weak initiating activity. In
neither case was this as strong as that of a molecularly equivalent dose of urethane.
(See Addendum II).

6. Four miscellaneous substances were tested. Of these 1,2-benzanthracene
was found to be an effective initiator of carcinogenesis but not carcinogenic, in
the doses used; and /-propiolactone was found to be an initiator of carcinogenesis.
The latter is being tested for carcinogenicity at present. (See Addendum I.)

7. The use of the " t " test of statistical significance for skew distributions,
such as those encountered in these results, is discussed. As an alternative, a
ranking test is proposed.

8. The histological appearance of the skin following one, or two, applications
of the test substances is described. Of those with initiating activity triethylene
melamine, ethyl N-methyl carbamate and ethyl N-phenyl carbamate gave rise
to no recognisable changes in the skin. 1,2-Benzanthracene consistently produced
a moderate epidermal hyperplasia. The response to fi-propiolactone varied from
slight to marked epidermal hyperplasia.

9. The results are discussed, and an attempt is made to correlate initiating
activity with other properties.

We are indebted to Dr. P. Armitage, of the Statistical Research Unit, London
School of Hygiene and Tropical Medicine, for valuable assistance in the mathe-
matical treatment of the results, and in particular for suggesting the use of
the ranking test for significance.

We wish to thank Professor E. Boyland, Mr. G. M. Timmis, and Dr. A. R.
Walpole for information about certain of the substances tested. Miss O. M.

200

STUDIES ON INCOMPLETE CARCINOGENESIS                   201]

Glendenning, Mr. W. J. Milton, Mr. J. A. Rawlings, and Mr. D. A. Woodcock
have given skilled technical assistance. The expenses of this research were partly
defrayed out of a block grant from the British Empire Cancer Campaign.

ADDENDUM

Since this paper was submitted for publication some of the experiments refered to
in the text as still in progress have been completed.

I: Further observations on 3.propiolactone. (cf. pp. 194, 198, 200.)

a. A group of 10 mice painted weekly for more than 20 weeks with a sub-ulcerative

concentration (0'3 ml. 2'5 per cent in acetone) of 3-propiolactone has so far
exhibited no skin tumours. In another group which received 10 similar
applications of f-propiolactone together with a standard course of croton oil
treatment, 108 papillomas were present on 7 of the 8 survivors at the end of treat-
ment. A further group received a single application of 2'5 per cent r-propio-
lactone followed after an interval of 3 weeks by a standard course of croton
oil. Five of the 9 survivors in this group had a total of 22 papillomas at the
end of treatment.

b. At post mortem only 1 lung adenoma was seen in 18 mice treated with P-propio-

lactone and croton oil.

Conclusion: There is no evidence that r-propiolactone applied in sub-ulcerative
concentration is carcinogenic for mouse skin, but its initiating action has been confirmed.

II: Further observations on ethyl N-phenylcarbamate (phenylurethane). (cf. pp. 191, 200.)

Twenty mice received 120 mg. phenylurethane (2 applications 20 per cent w/v in
acetone with 15 minutes interval) weekly for 7 weeks, and 60 mg. weekly for a further
8 weeks. Three days after the first application of phenylurethane a standard course of
croton oil was begun. At the end of treatment only 1 of the 16 survivors bore tumours
(5 papillomas), an incidence not differing significantly from that in mice receiving croton
oil only.

A further group which received similar treatment with phenylurethane but no croton
oil developed no skin tumours.

Conclusion: This test has failed to confirm the suggestion that phenylurethane
possesses weak initiating activity, and has shown that it is not carcinogenic for mouse
skin in the doses tested.

REFERENCES.

ANDERVONT, H. B. AND SHIMKIN, M. B.-(1940) J. nat. Cancer Inst., 1, 225.
ARNON, D. I. AND WHATLEY, F. R.-(1949) Arch. Biochem., 23, 141.

BADGER, G. M.-(1954) 'Advances in Cancer Research'. New York (Academic Press

Inc.), pp. 73-127.

Idem ELSON, L. A., HADDOW, A., HEWETT, C. L. AND ROBINSON, A. M.-(1942) Proc.

Roy. Soc., B, 130, 255.

BARRY, G., COOK, J. W., HASLEWOOD, G. A. D., HEWETT, C. L., HIEGER, I. AND KENNA-

WAY, E. L.-(1935) Ibid., 117, 318.

BAYRD, E. D., STICKNEY, J. M., HALL, B. E. AND WATKINS, C. H.-(1952) Cancer, 5,336.
BERENBLUM, I.-(1935) J. Path. Bact., 40, 549.-(1941) Cancer Res., 1, 807.-(1951)

J. nat. Cancer Inst., 11, 839.

BERMAN, L. AND AXELROD, A. R.-(1948) Amer. J. clin. Path., 18, 104.

202                  F. J. C. ROE AND M. H. SALAMAN

BOYLAND, E.-(1954) Oncologia, 7, 144.

Idem AND HORNING, E. S.-(1949) Brit. J. Cancer, 3, 118.

BUTRCHENAL, J. H., CROSSLEY, M. L., STOCK, C. C. AND RHOADS, C. P.-(1950) Arch.

Biochem., 26, 321.

COOK, J. W.-(1933) Congr. int. Cancer, 2, 373.

CORNMAN, I.-(1947) J. exp. Biol., 23, 292.-(1954) 'International Review of Cytology',

New York (Academic Press Inc.), Vol. III, pp. 113-130.
DIXON, W. E. AND MALDEN, W.-(1908) J. Physiol., 37, 50.
DUSTIN, A. P.-(1929) Arch. Anat. micr., 25, 37.

Idem AND GREGOIRE, M. CH.-(1933) Bull. Acad. Ml. Belg., 13, 585.
Idem AND PITON, R. (1929) Ibid., 9, 26.

DUSTIN, P.-(1947a) Nature, 159, 794.-(1947b) Brit. J. Cancer, 1, 48.
EADIE, G. S.-(1942) J. biol. Chem., 146, 85.

ENGSTROM, R. M., KIRSCHBAUM, A. AND MIXER, H. W.-(1947) Science, 105, 255.
GAFFRON, H. AND FAGER, E. W.-(1951) Annu. Rev. P1. Physiol., 2, 87.

GAENSLER, E. A., MCKAY, D. G., WARE, P. F. AND LYNCH, J. P.-(1948) Arch. Path., 46,

503.

GALTON, D. A. G.-(1953) Lancet, i, 208.

GELLHORN, A.-(1953) Cancer Res., 13, 205.

GOODMAN, L. S., WINTROBE, M. M., DAMESHEK, W., GOODMNAN, M. J., GILMAN, A. AND

MCLENNAN, M. T.-(1946) J. Amer. med. Ass., 132, 126.
GRAFFI, A.-(1953) Schweiz. med. Wschr., 83, 865.

Idem, VLAMYNCH, E., HOFFMAN, F. AND SCHULTZ, I.-(1953) Arch. Geschwulstforsch., 5,

110.

GRIFFIN, A. C., BRANDT, E. L. AND TATUM, E. L.-(1951) Cancer Res., 11, 253.
GWYNN, R. H. AND SALAMAN, M. H.-(1953) Brit. J. Cancer, 7, 482.

HADDOW, A.-(1935) NVature, 136, 868.-(1938) J. Path. Bact., 47, 567.-(1950) Ann.

Rep. Brit. Emp. Cancer Campgn., 28, 56.

Idem AND HORNING, E. S.-(1950) J.R. micr. Soc., 70, 181.

Idem AND ROBINSON, A. M.-(1937) Proc. Roy. Soc., B, 122, 442.-(1939) Ibid., 127, 277.
Idem, SCOTT, C. M. AND SCOTT, J. D.-(1937) Ibid., 122, 477.

Idem AND TIMMIS, G. M.-(1951) Acta Un. int. Cancr., 7, 469.-(1953) Lancet, i, 207.
HAMPERL, H.-(1946) Klin. u. Praxis, 1, 186.
HARDE, E.-(1939) Congr. int. Cancer, p. 126.

HENDRY, J. A., HOMER, R. F., ROSE, F. L. AND WALPOLE, A. L.-(1951) Brit. J.

Pharmacol, 6, 357.

HESTON, W. E., LORENZ, E., AND DERINGER, M. K.-(1953) Cancer Res., 13, 573.

HILL, W. T., STANGER, D. W., PIZZO, A., RIEGEL, B., SHUBIK, P. AND WARTMAN, W. B.

-(1951) Ibid., 11, 892.

HUGHES, A. F. W.-(1950) Quart. J. micr. Sci., 91, 251.

JOHNSON, F. H., EYRING, H. AND KEARNS, W.-(1943) Arch. Biochem., 3, 1.
JONES, R. N.-(1940) J. Amer. chem. Soc., 62, 148.
KENNAWAY, E. L.-(1930) Biochem, J., 24, 497.
KING, L. S.-(1948) J. nat. Cancer Inst., 8, 215.

Idem AND SULLIVAN, M.-(1946) Science, 104, 244.

KNEEDLER, W. H.-(1945) J. Amer. med. Ass., 129, 272.

KOFFLER, H. AND MARKERT, I. L.-(1951) Proc. Soc. exp. Biol. N. Y., 76, 90.
KOLLER, P.-(1953) Heredity, supplement to Vol. 6, 181.

KRUTSKAL, W. H. AND WALLIS, W. A.-(1952) J. Amer. statist. Ass., 47, 583.

LARSEN, C. D.-(1947a) Cancer Res., 7, 726-(1947b) J. nat. Cancer Inst., 8, 99.-(1948)

Ibid., 9, 35.

Idem, RHOADS, P. B. Jr. AND WEED, L. L.-(1946) Ibid., 7, 5.
LASNITZKI, I.-(1949) Brit. J. Cancer, 3, 501.

LEFEVRE, J.-(1939) C.R. Acad. Sci., Paris, 208, 301.

STUDIES ON INCOMPLETE CARCINOGENESIS                    203

LEWIS, M. R. AND CROSSLEY, M. L.-(1950) Arch. Biochem., 26, 319.
LOVELESS, A. AND REVELL, S.-(1949) Nature, 164, 938.

LUDFORD, R. J.-(1936) Arch. exp. Zellforsch, 18, 411.-(1953) J.R. micr. Soc., 73, 1.
MICHAELIS, M. AND QUASTEL, J. H.-(1941) Biochem J., 35, 518.
MOTTRAM, J. C. AND DONIACH, I.-(1938) Lancet, i, 1156.

NARPOZZI, A.-(1953) Riv. Anat. pat. Oncol (Padova), 6, 1155.

NETTLESHIP. A. AND HENSHAW, P. S.-(1943) J. nat. Cancer Inst., 4, 309.
ORR, J. W.-(1938) J. Path. Bact., 46, 495.
OSTERGREN, G.-(1948) Bot. Notiser, 4, 376.

PATERSON, E., HADDOW, A., THOMAS, J. A. AND WATKINSON, J. M.-(1946) Lancet, i,

677.

Idem, KUNKLER, P. B. AND WALPOLE, A. L.-(1953) Brit. med. J., i, 59.
PETERING, H. G.-(1952) Physiol. Rev., 32, 197.

PETRAKIS, N. L., BIERMAN, H. R., KELLY, K. H., WHITE, L. P. AND SHIMKIN, M. B.-

(1954) Cancer, 7, 383.

PITON, R.-(1929) Arch. int. M&d. exp., 5, 355.

QUASTEL, J. H. AND WHEATLEY, A. H. M.-(1933) Proc. Roy. Soc., B, 112, 60.-(1934)

Biochem. J., 28, 1521.

ROE, F. J. C., AND SALAMAN, M. H.-(1954) Brit. J. Cancer, 8, 666.

ROSE, F. L., HENDRY, J. A. AND WALPOLE, A. L.-(1950) Nature, 165, 993.
SALAMAN, M. H. AND GWYNN, R. H.-(1951) Brit. J. Cancer, 5, 252.
Idem AND ROE, F. J. C.-(1953) Ibid., 7, 472.

SHEAR, M. J. AND LEITER, J.-(1941) J. nat. Cancer Inst., 2, 241.
SHIMKIN, M. B.-(1954) Cancer, 7, 410.

SIMONET, M., AND GUINOCHET, M.-(1939) C.R. Soc. Biol., Paris, 131, 222.
SKIPPER, H. E. AND BRYAN, C. E.-(1949) J. nat. Cancer Inst., 9, 391.

lidem, RISER, W. H., Jr., WELTY, M. AND STELZENMULLER, A.-(1948) J. nat. Cancer

Inst., 9, 77.

SLYE, M., HOLMES, H. F. AND WELLS, H. G.-(1921) J. Cancer Res., 6, 57.
SMITH, H. H. AND SRB, A. M.-(1951) Science, 114, 490.

STEDMAN, E. AND STEDMAN, E.-(1931) Biochem. J., 25, 1147.-(1932) Ibid., 26, 1214.
STEINER, P. E. AND FALK, H. L.-(1951) Cancer Res., 11, 56.

THOMSON, W.-(1930a) J. Hyg., Camb., 36, 24.-(1930b) Ibid., 36, 156.
VASIC, V.-(1953) Mfg. Chem., 24, 197.

WALPOLE, A. L., ROBERTS, D. C., ROSE, F. L., HENDRY, J. A. AND HOMER, R. F.

(1954) Brit. J. Pharmacol., 9, 306.

WARBURG, O. AND LUTTGENS, W.-(1944) Naturwiss. enschaften, 32, 161.

WILKINSON, J. F., HADDOW, A. AND NABARRO, J. D.-(1953) Proc. R. Soc. Med., 46, 685.
ZYLBERSZAC, S.-(1939) Acta brev. neerl. Physiol, 9, 240.

				


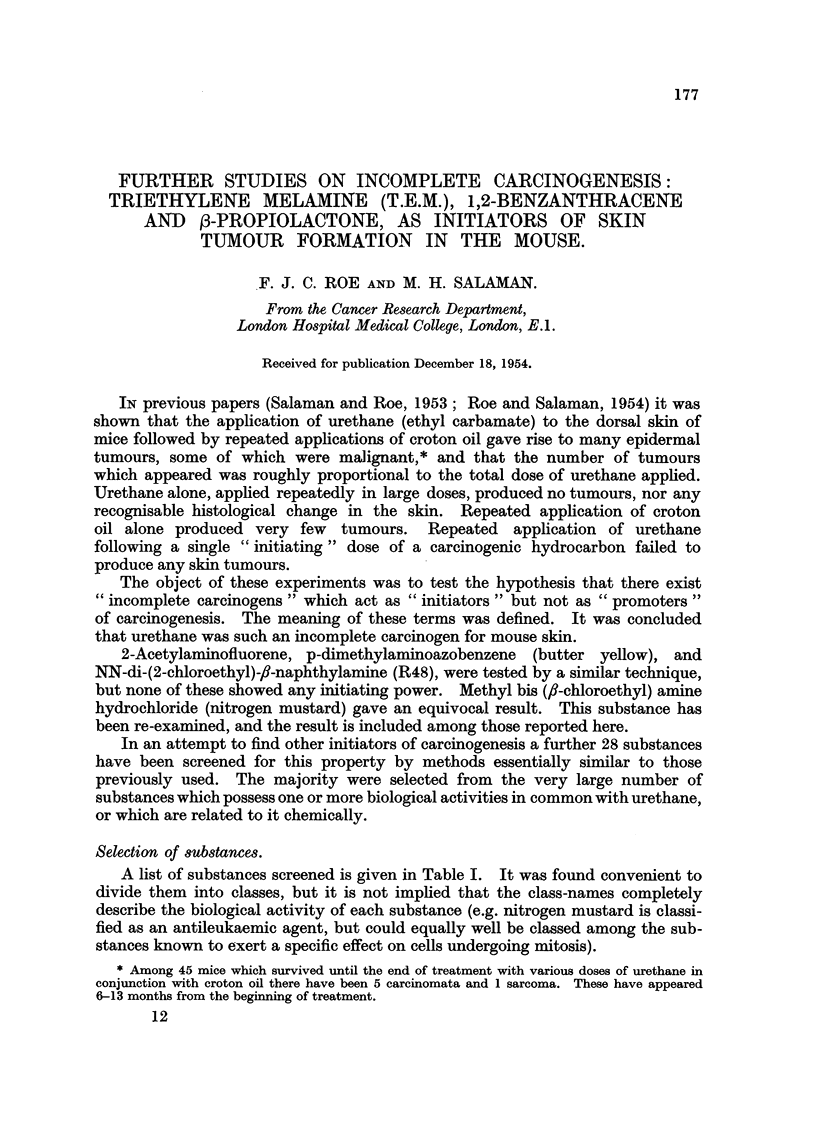

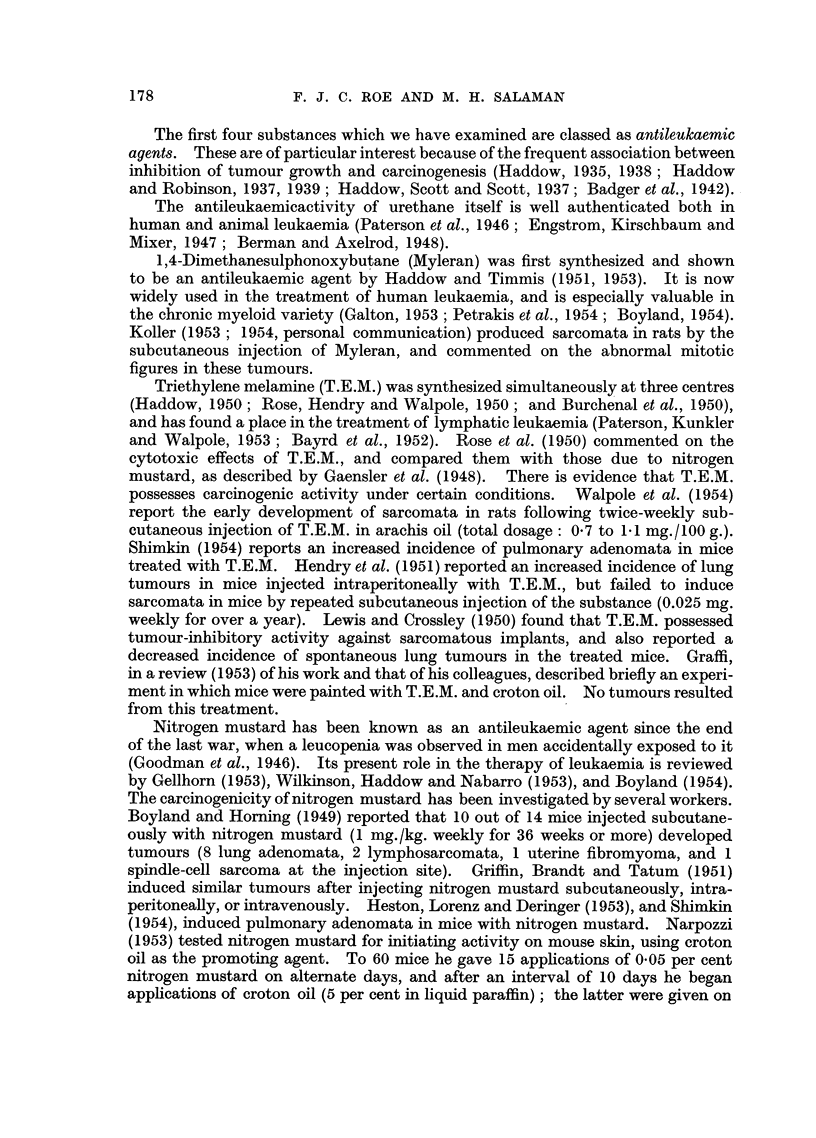

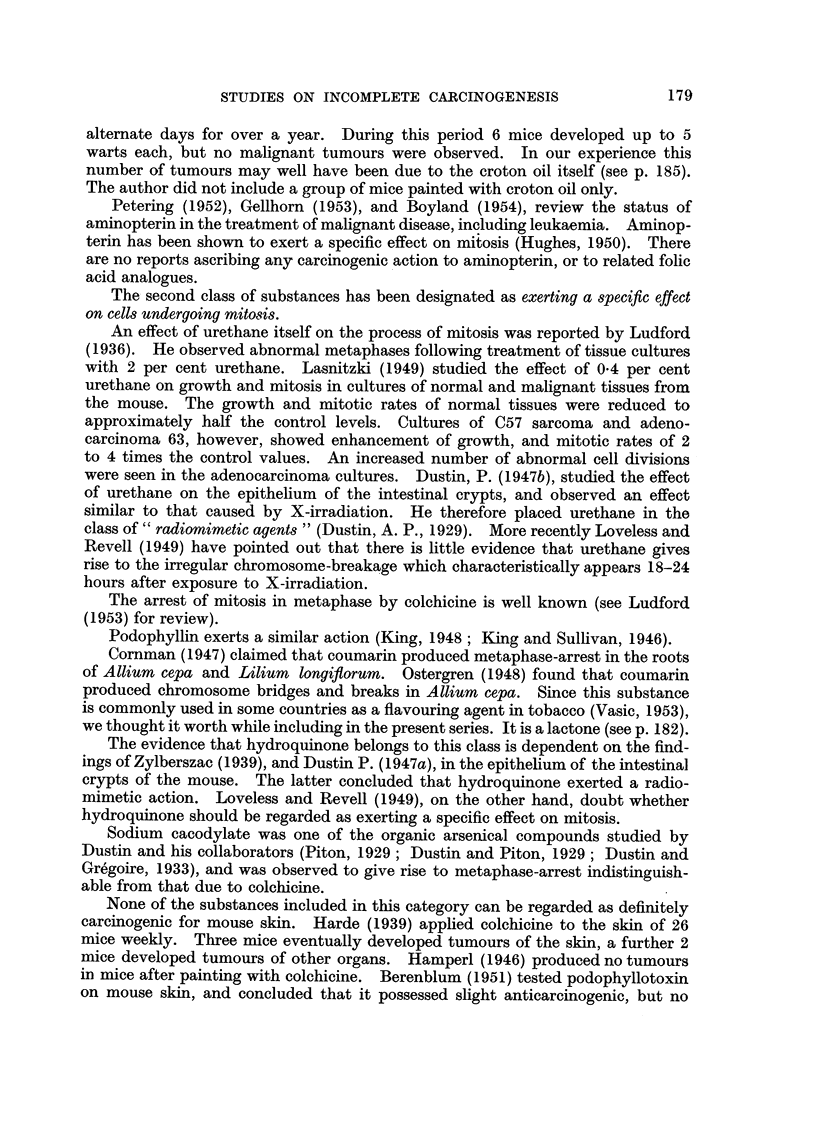

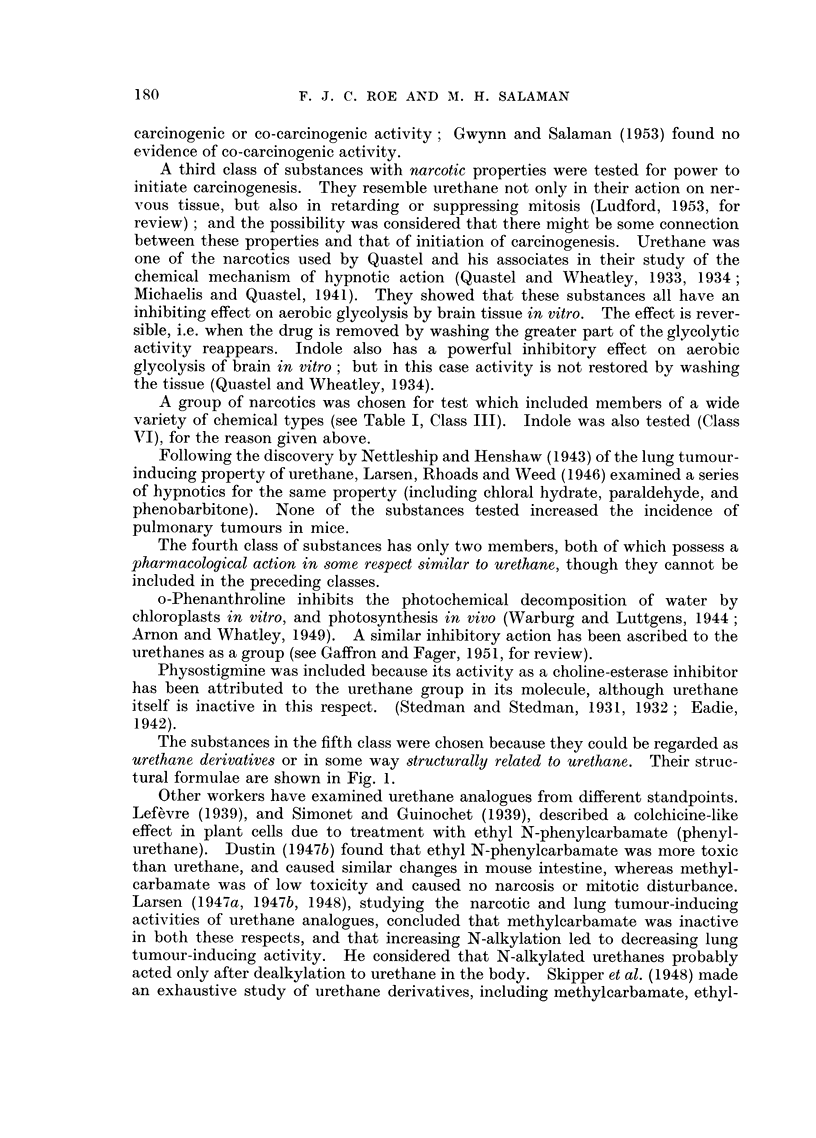

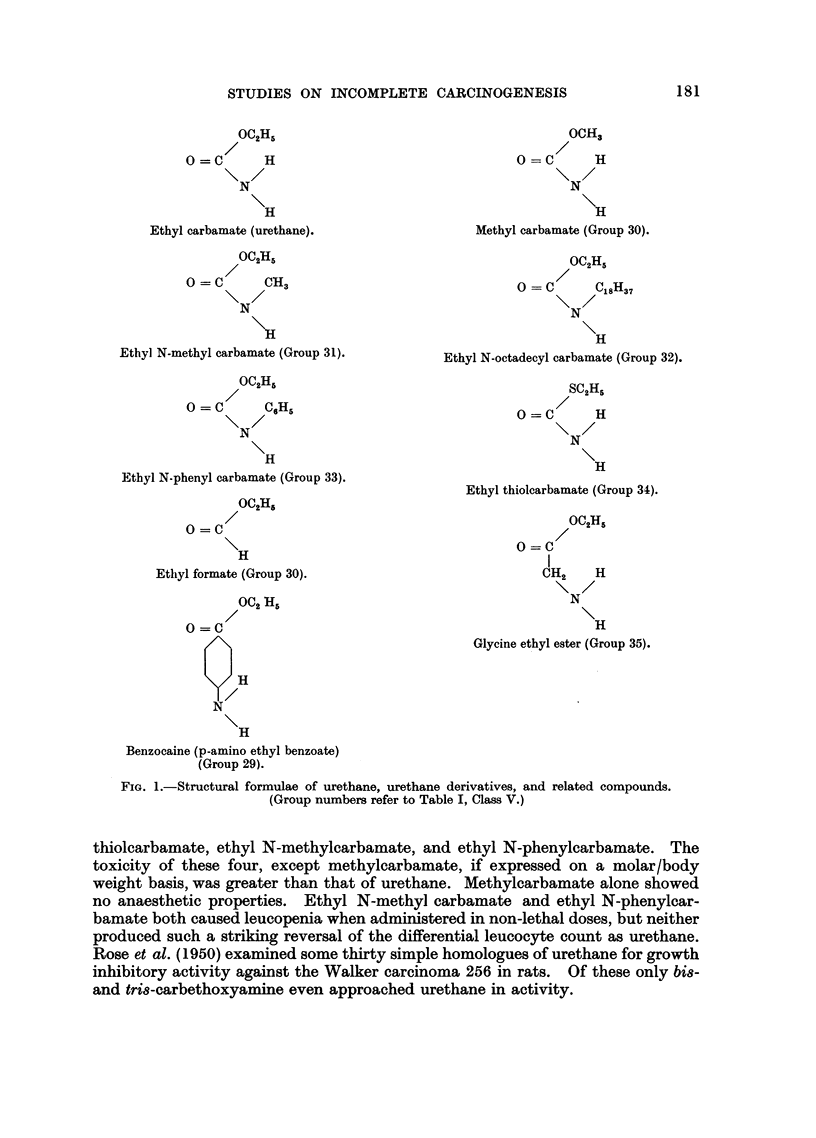

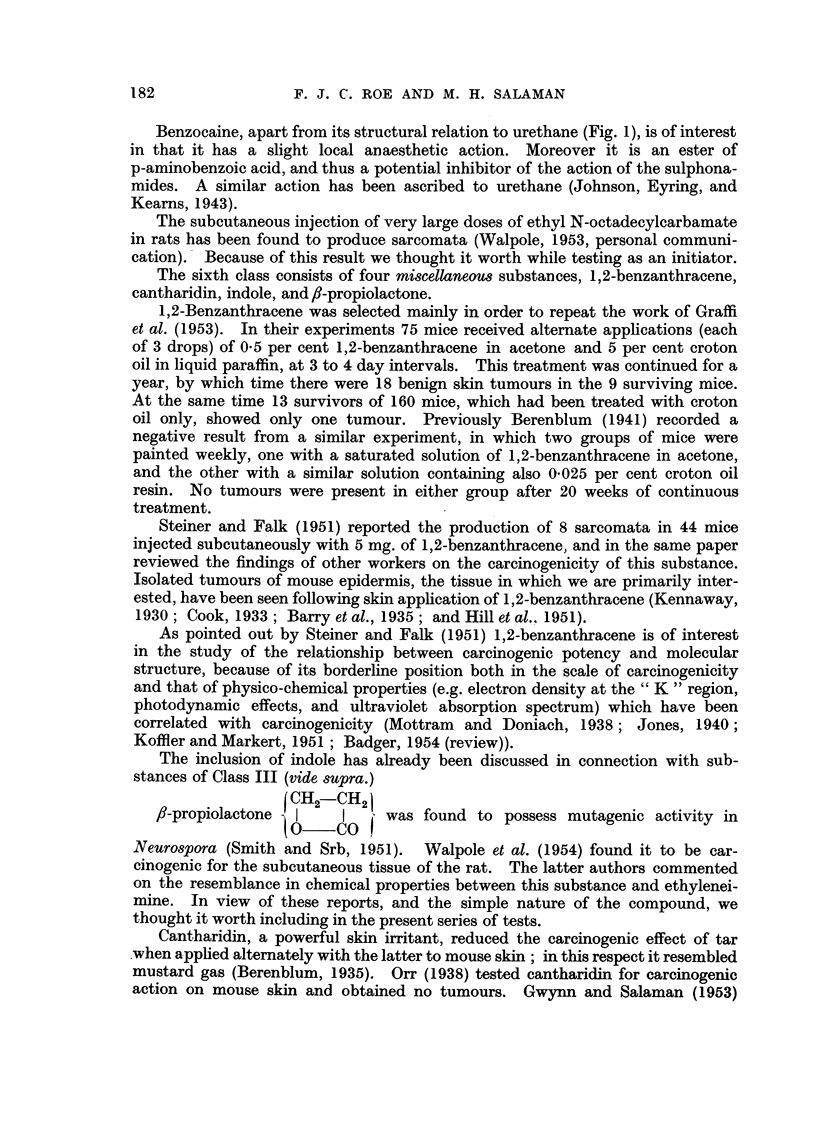

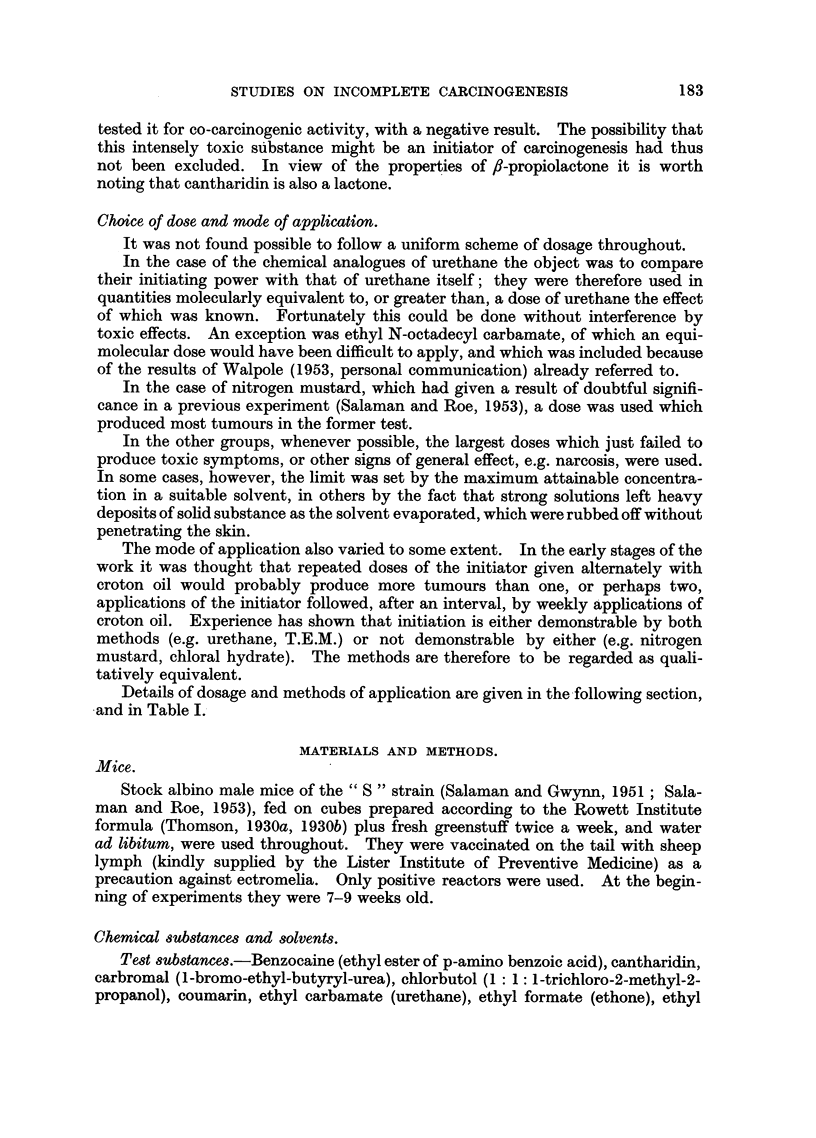

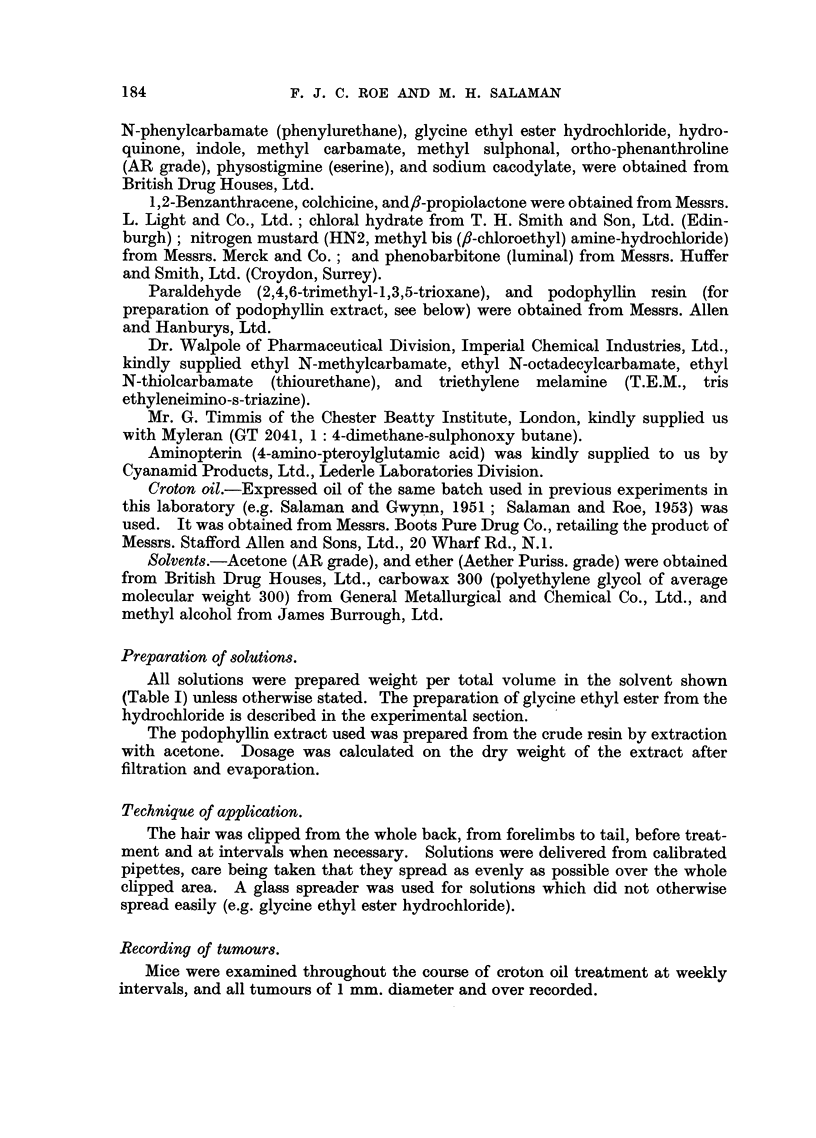

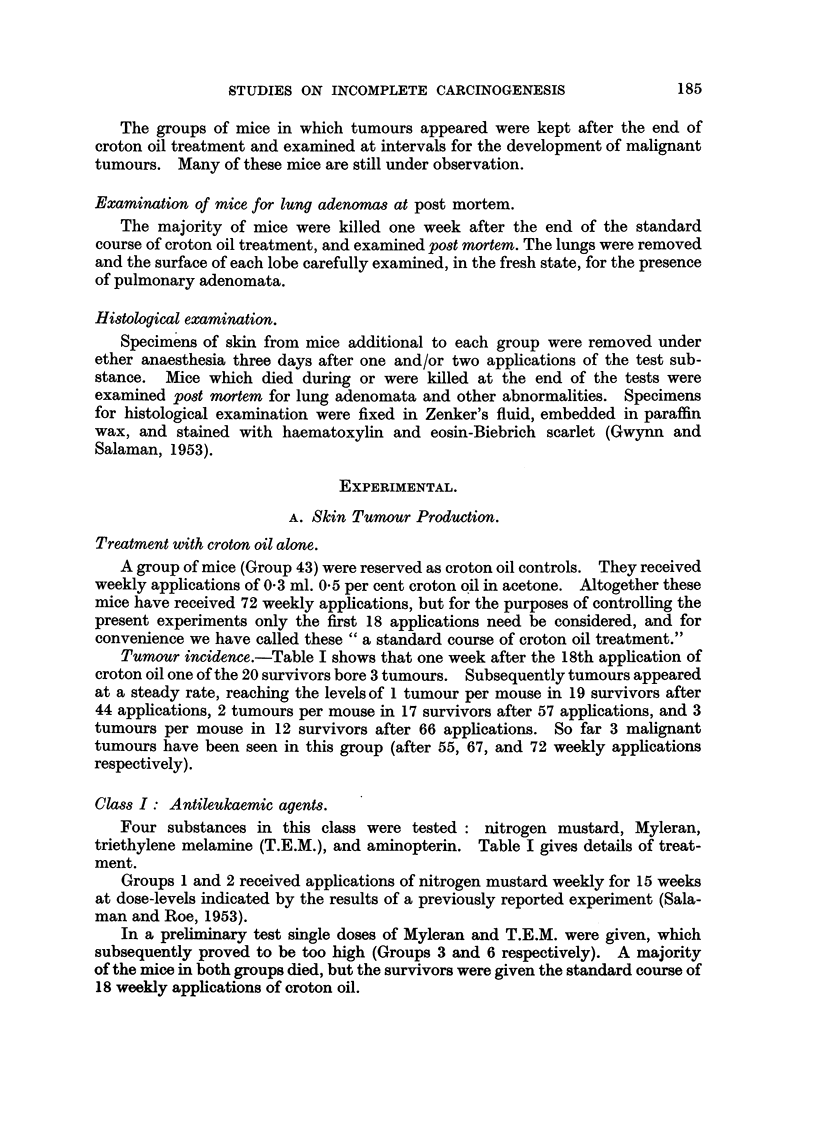

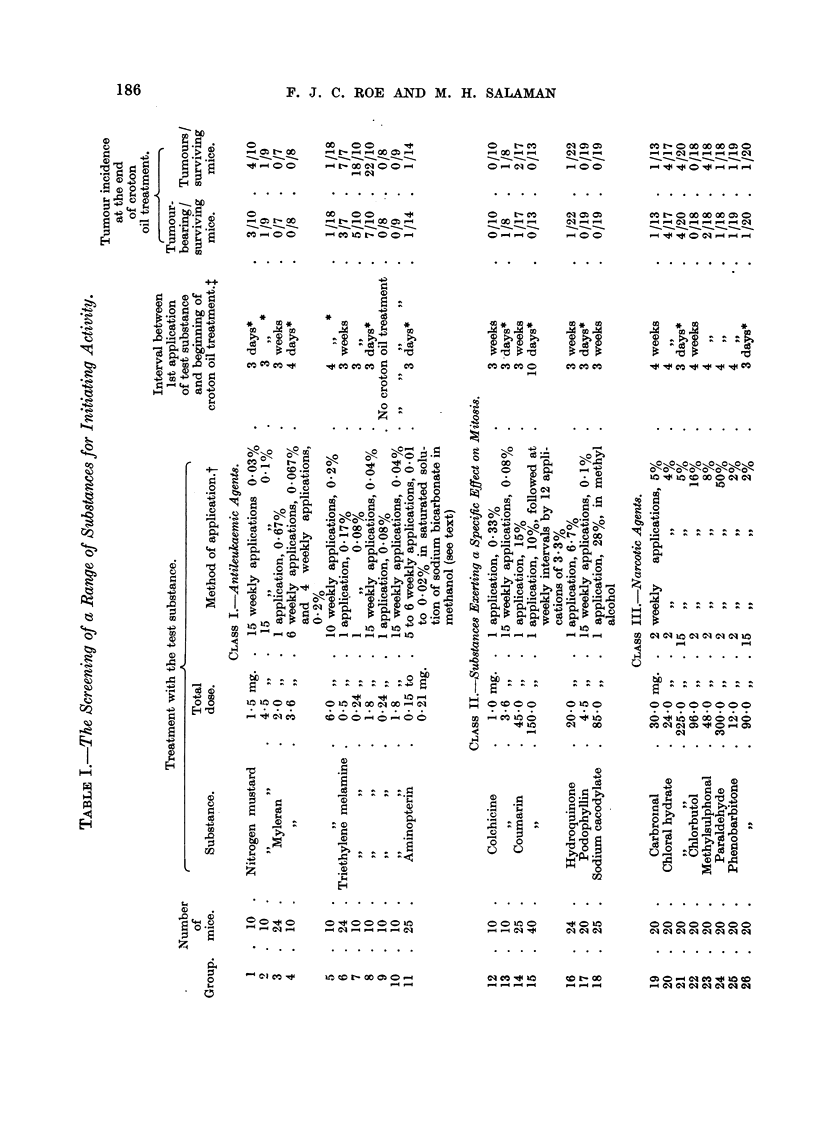

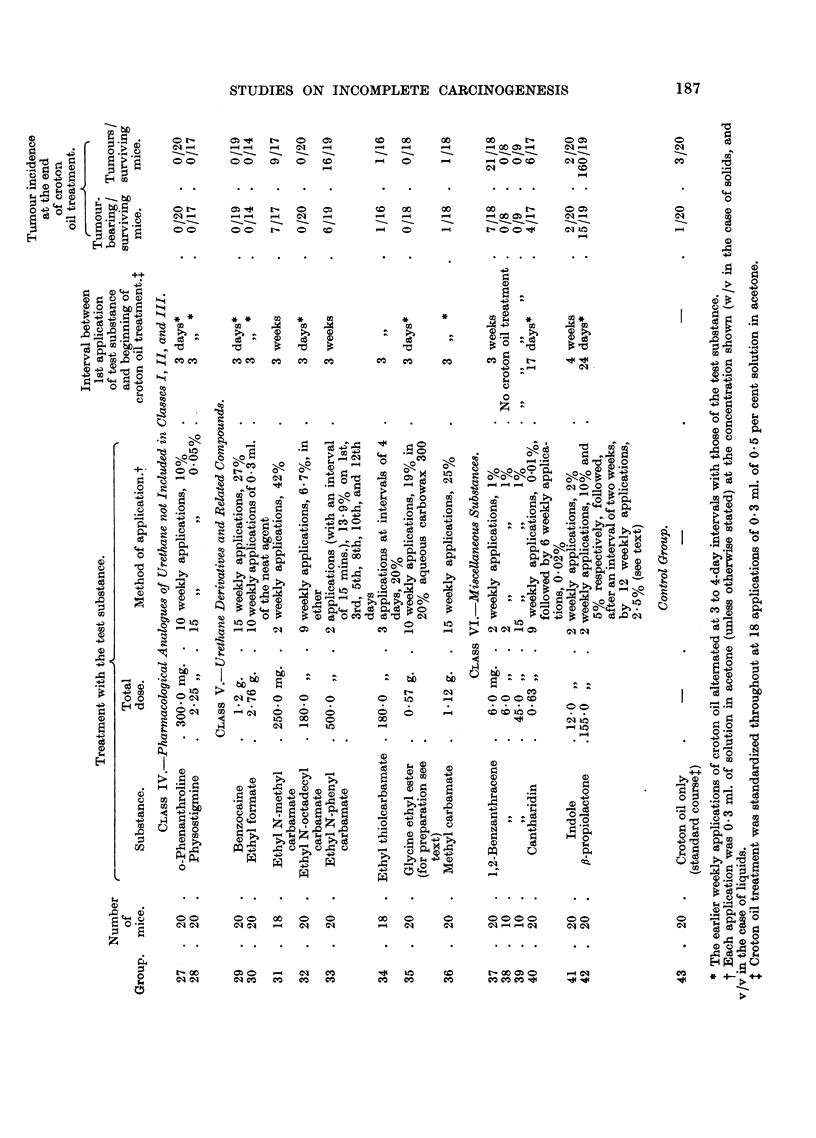

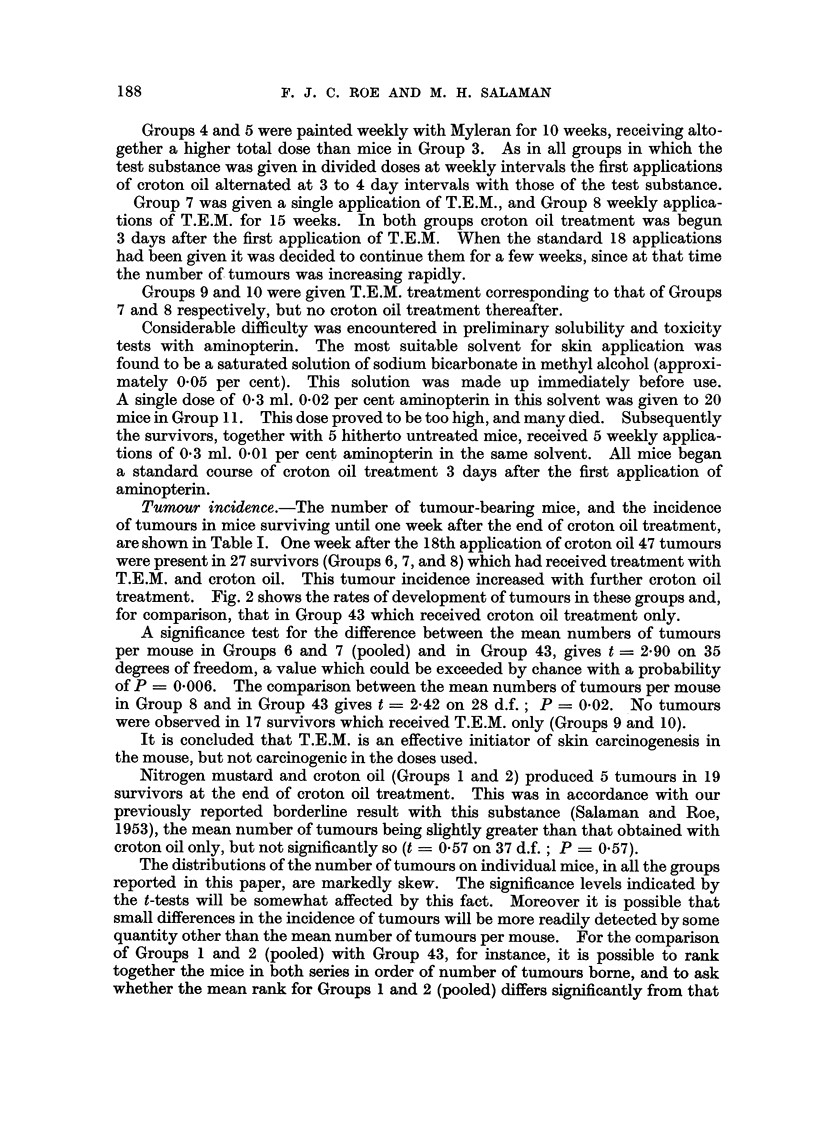

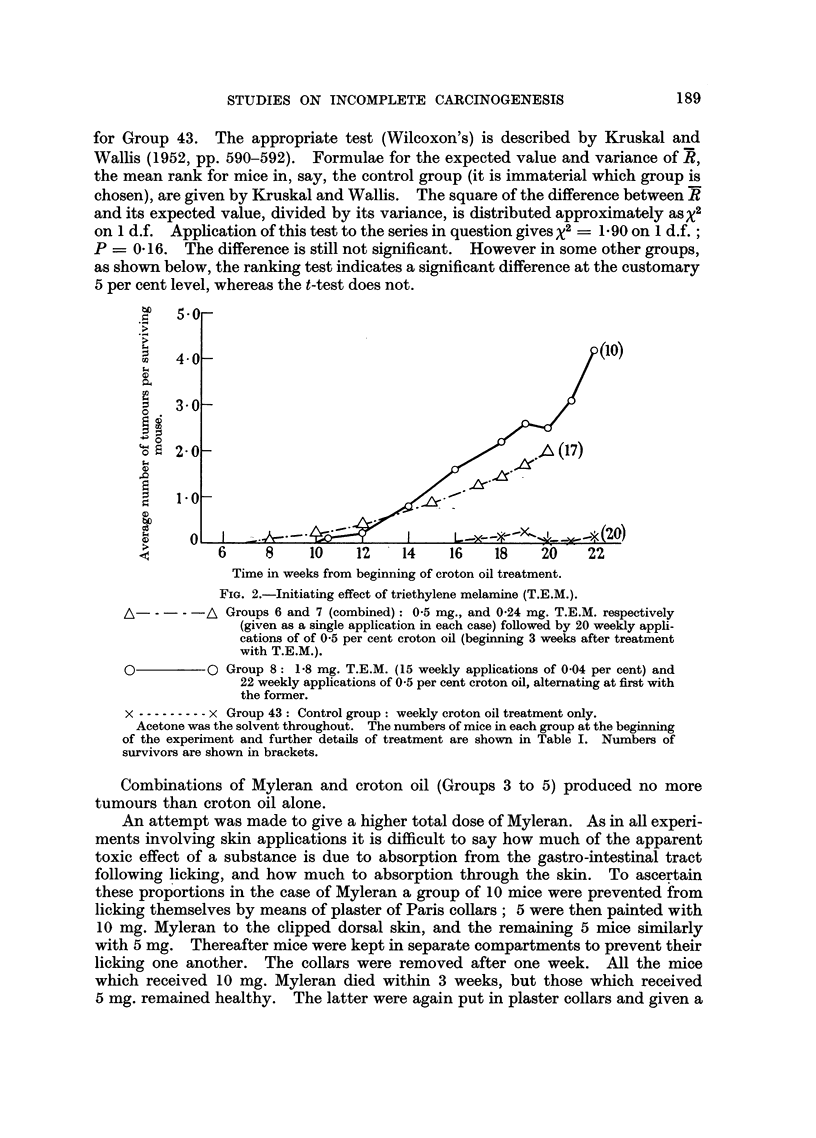

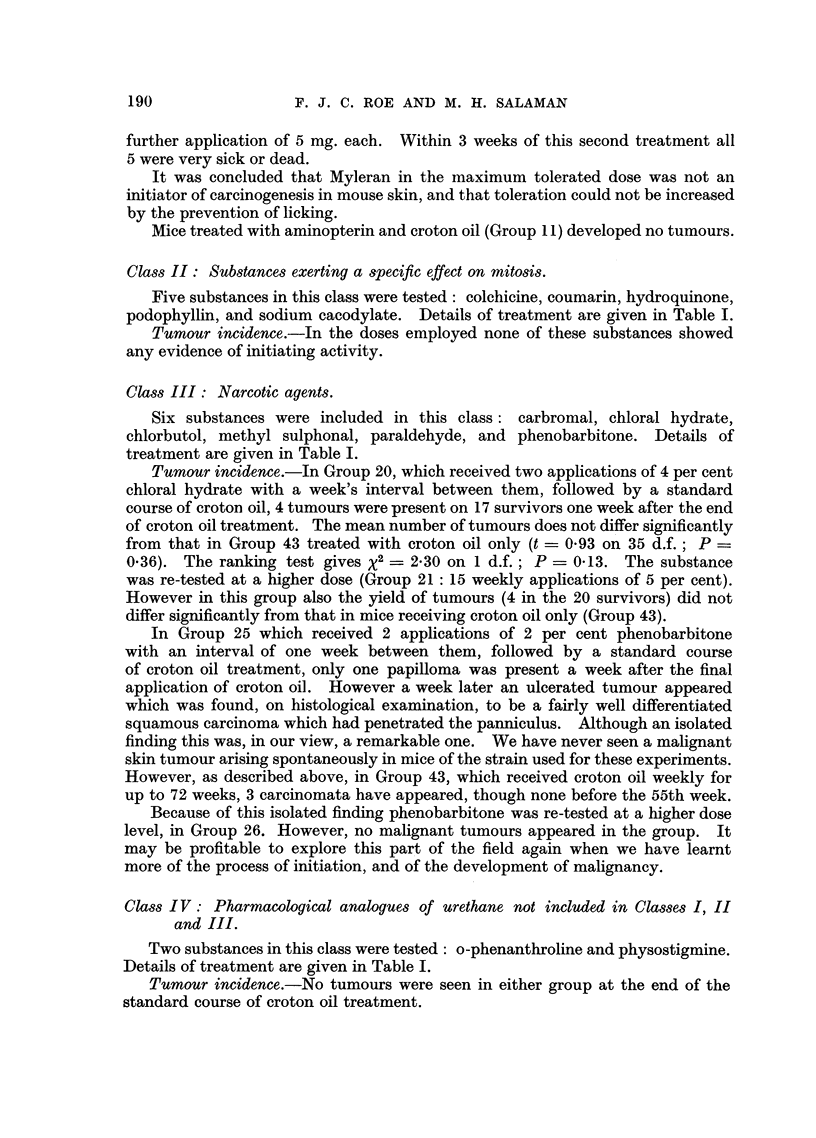

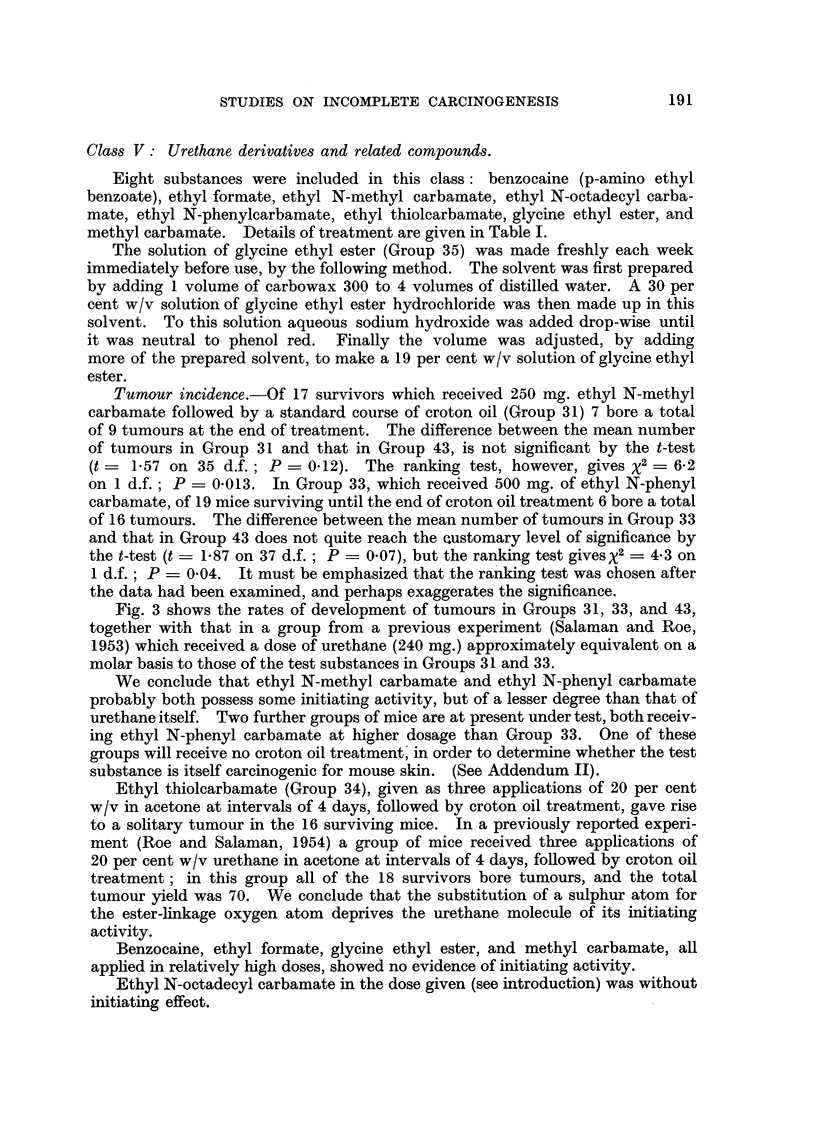

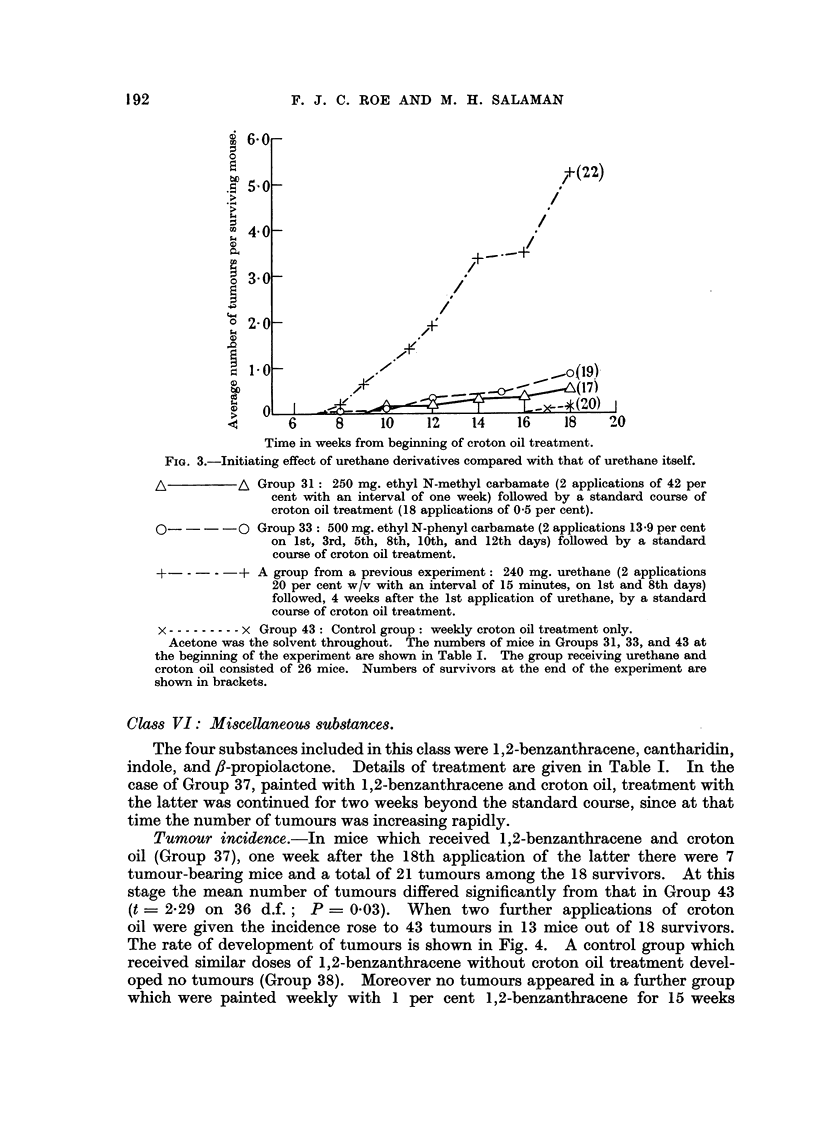

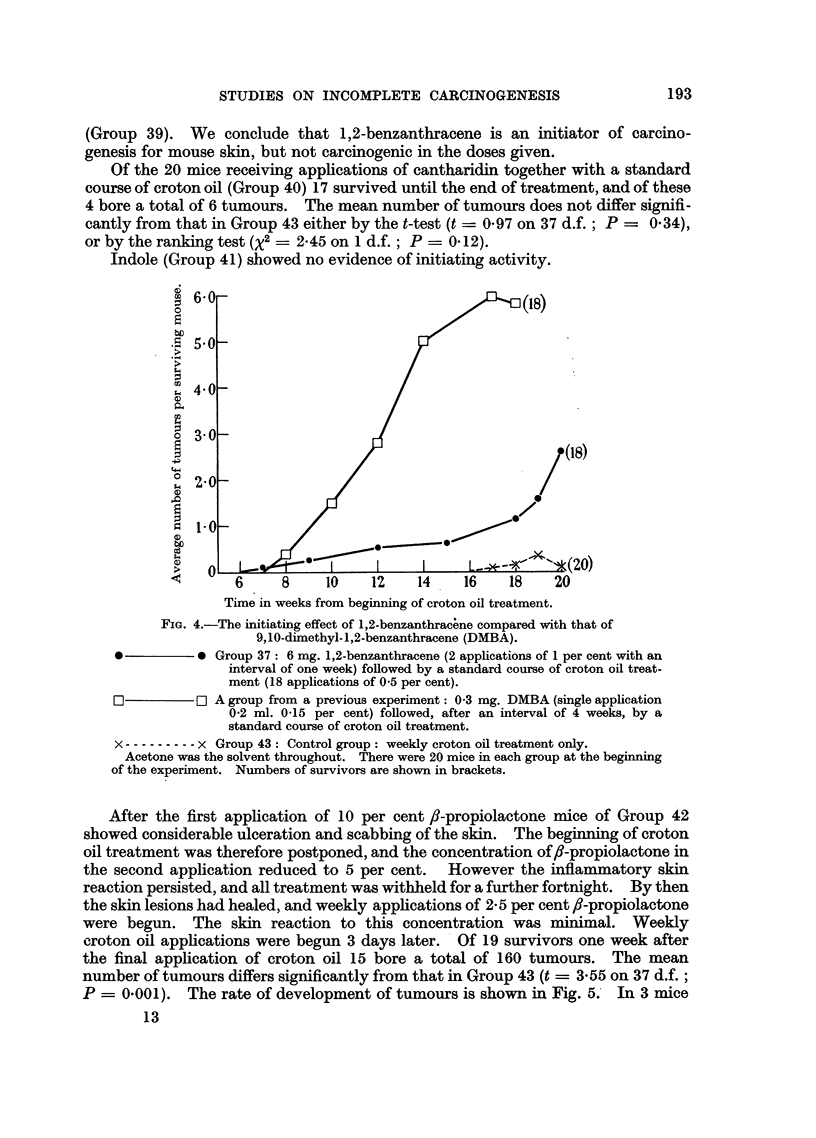

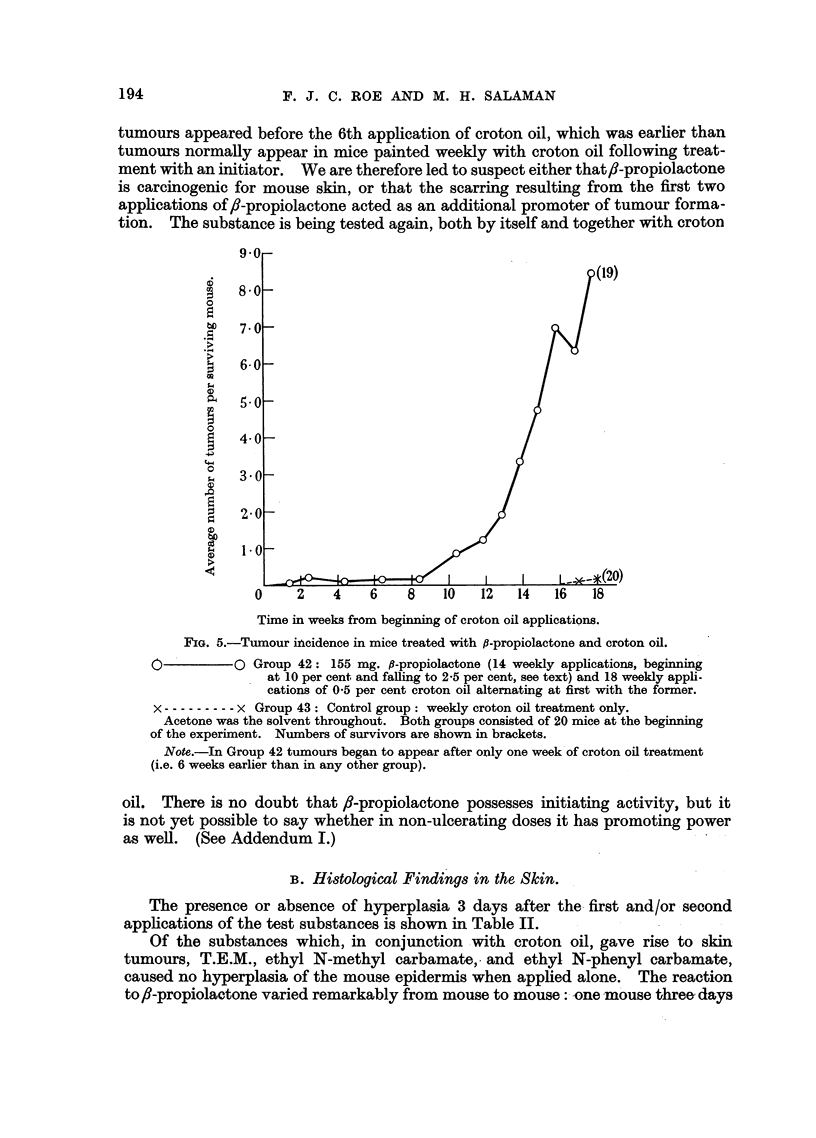

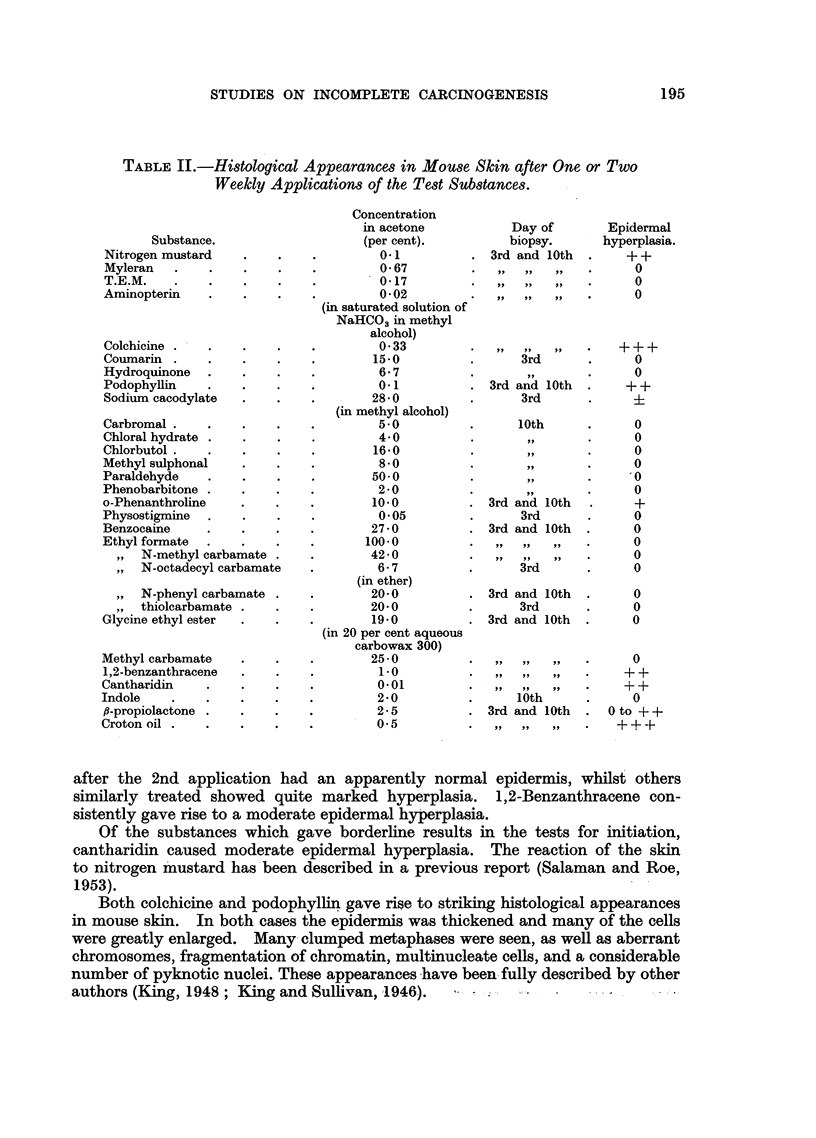

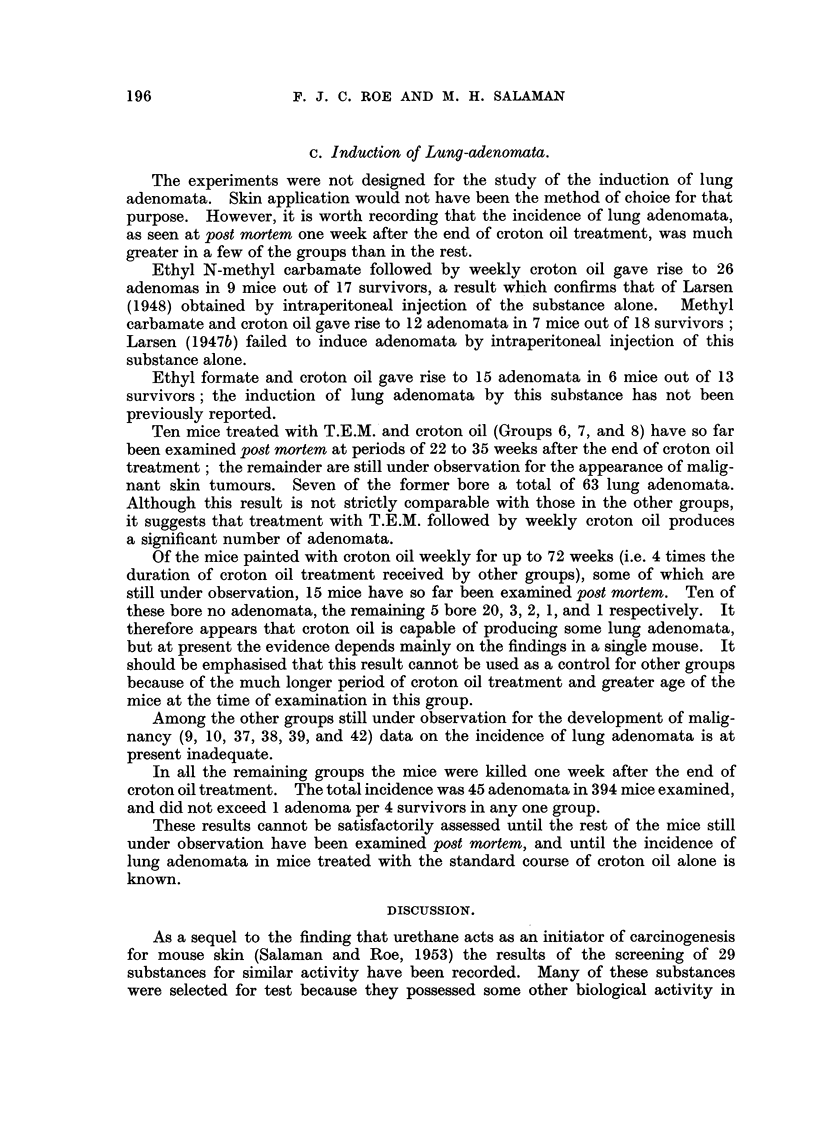

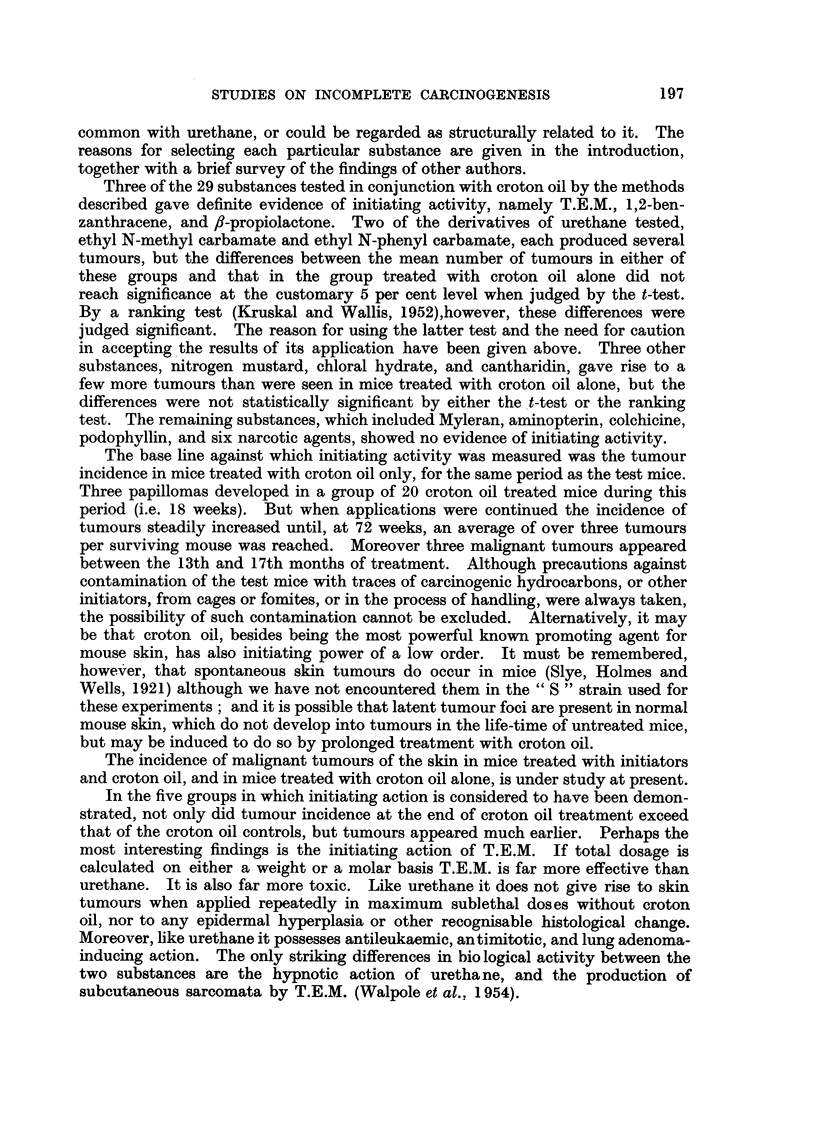

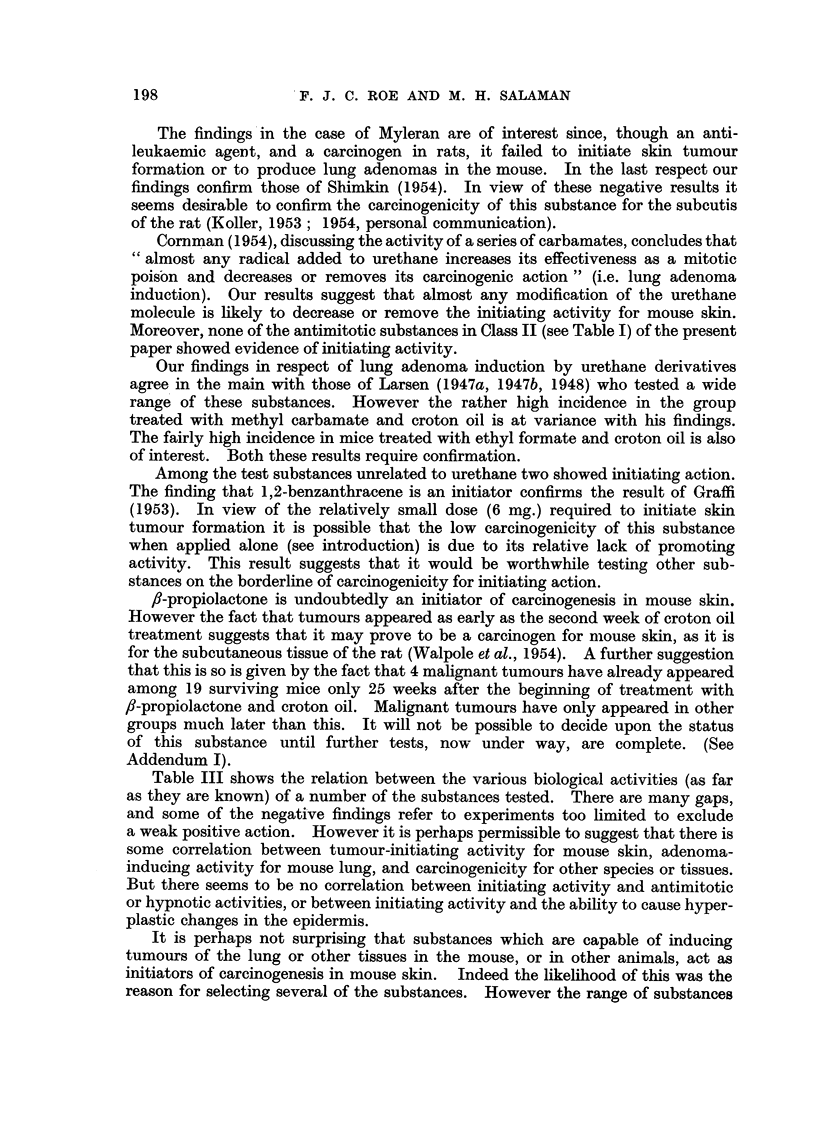

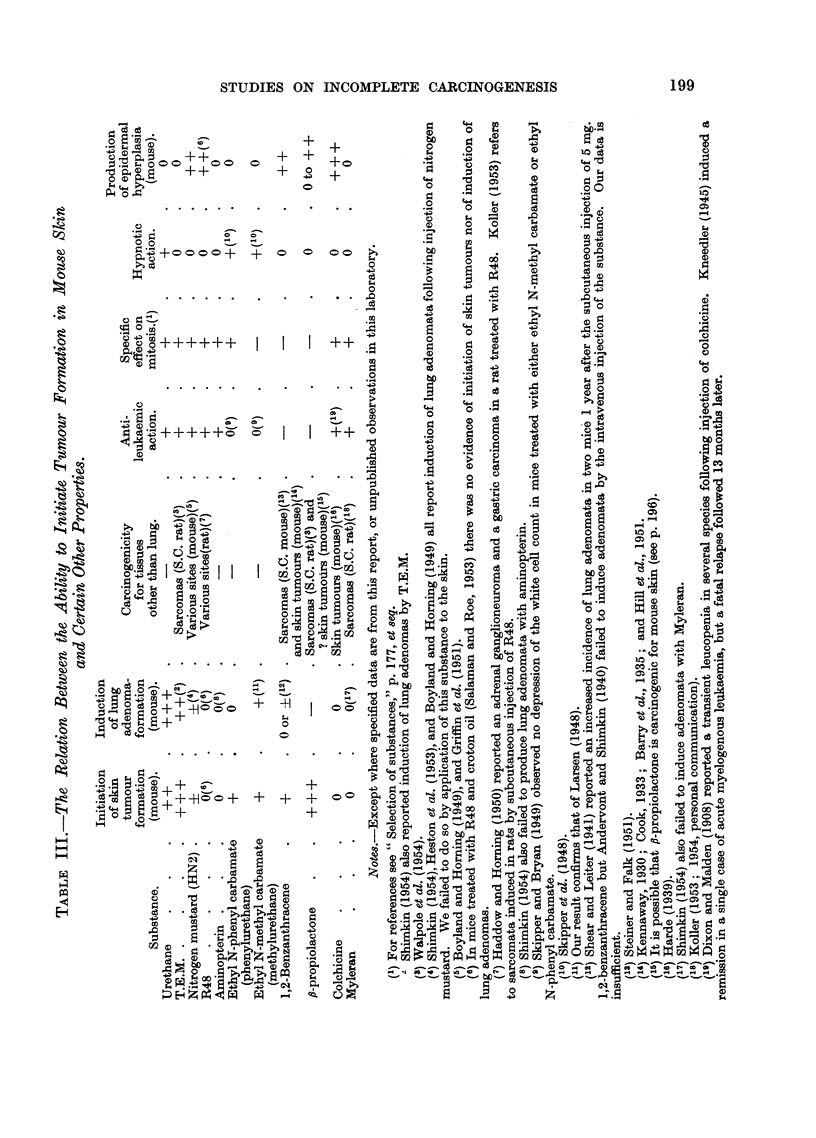

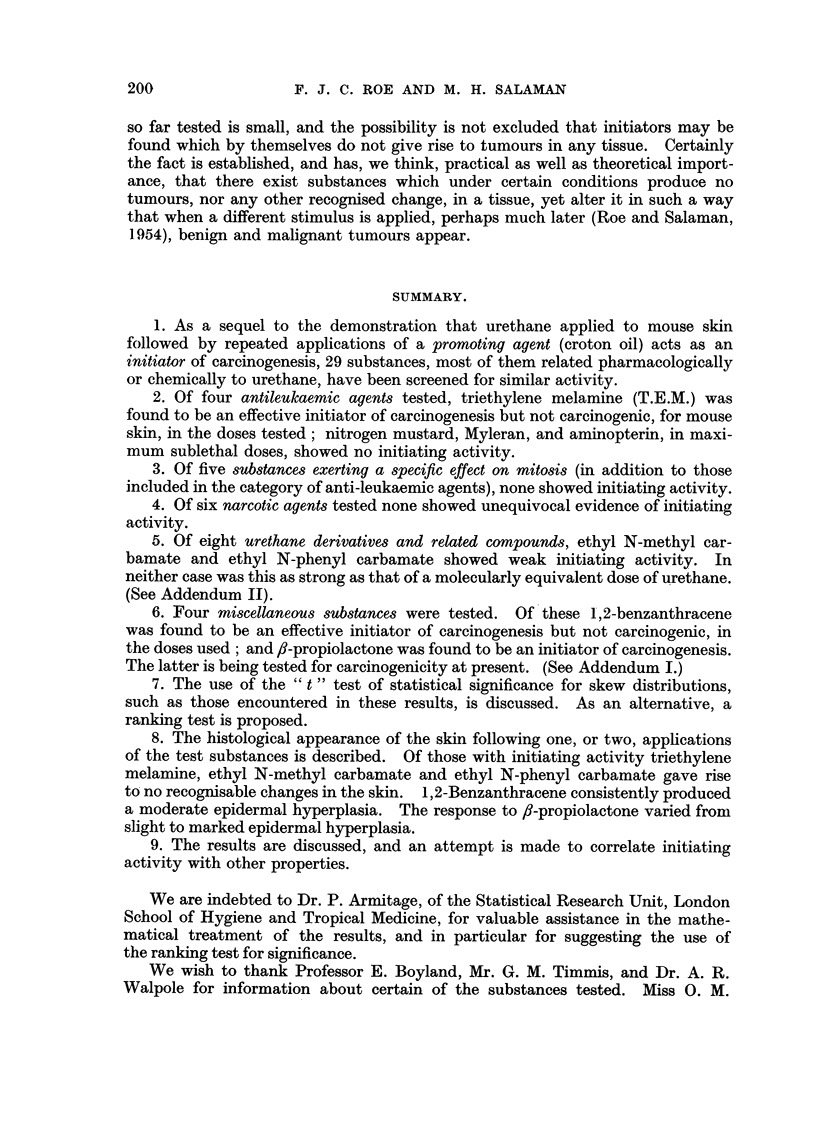

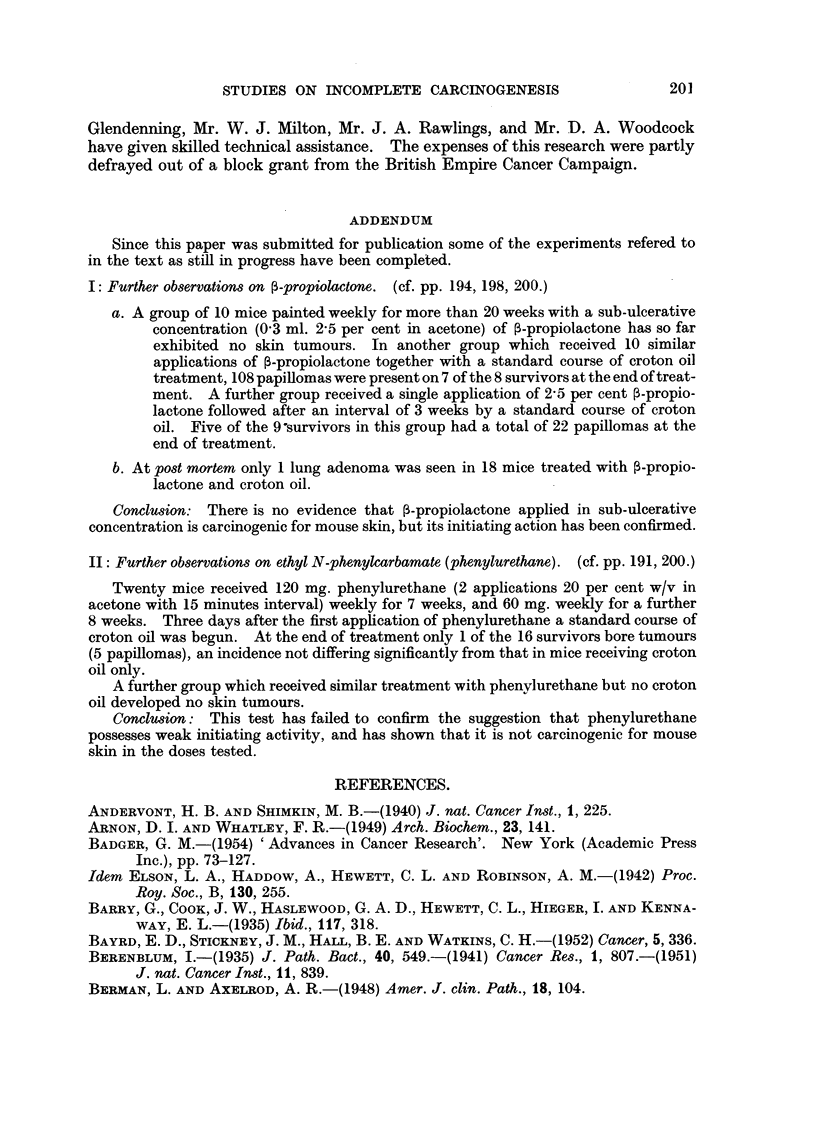

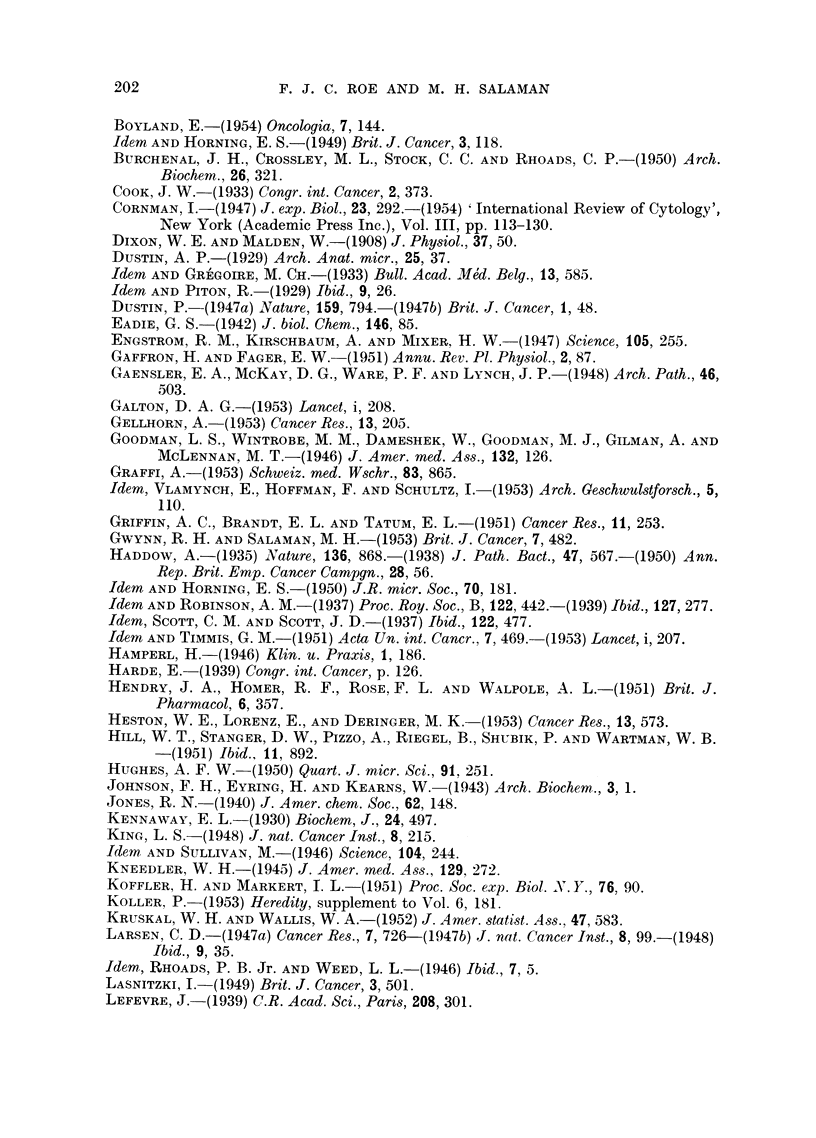

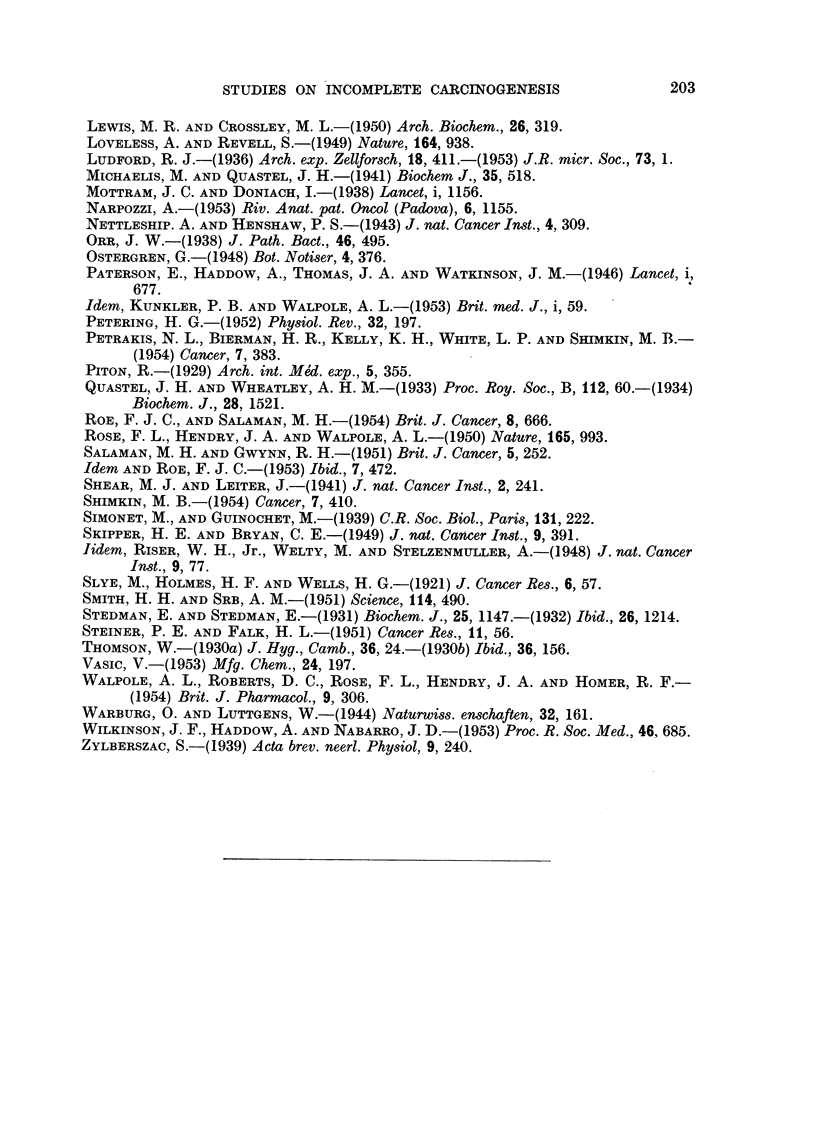

